# Comparison of right- and left-approach esophagectomy for esophageal cancer: a meta-analysis

**DOI:** 10.3389/fonc.2025.1685103

**Published:** 2026-01-23

**Authors:** Xiangdong Peng, Jiwen Luo, Jie Ren, Wen Liu, Banglin Xia, Guixian Liu

**Affiliations:** Department of Cardiothoracic Surgery, Mianyang Central Hospital, Mianyang, Sichuan, China

**Keywords:** esophageal cancer, Ivor-Lewis, McKeown, meta-analysis, sweet

## Abstract

**Objective:**

This study aims to compare the effects of left and right thoracic approaches on patients undergoing esophagectomy.

**Methods:**

A search was conducted across PubMed, Embase, Cochrane, and Web of Science for randomized controlled trials, cohort studies, and non-randomized trials that evaluated the effects of the two approaches on patients with esophageal cancer up to March 19, 2025. Two reviewers independently screened the retrieved articles, extracted relevant data, and appraised the risk of bias. A meta-analysis was performed using Stata statistical software.

**Results:**

A total of 21 studies were included. Compared with the left thoracic approach, the right approach had a longer surgical duration (mean difference [MD] = 77.51, 95% confidence interval [CI]: 53.19–101.84, P < 0.05), a higher number of lymph nodes removed (MD = 3.00, 95% CI: 0.30–5.69, P < 0.05), and a higher risk of anastomotic fistula (MD = 2.07, 95% CI: 1.49–2.88, P < 0.05), wound infection (MD = 1.68, 95% CI: 1.04–2.73, P < 0.05) and pulmonary complications (risk ratio = 1.39, 95% CI: 1.15–1.68, P < 0.01). There were no significant differences in the risk of chylothorax, postoperative hospital stays, long-term disease-free survival, or overall survival.

**Conclusion:**

Esophagectomy through the right thoracic approach achieves more thorough lymph node dissection, but it is associated with an increased risk of longer surgical duration, anastomotic fistula, wound infection, and pulmonary complications.

**Systematic Review Registration:**

https://www.crd.york.ac.uk/PROSPERO/view/CRD420251026319, identifier CRD420251026319.

## 1 Introduction

Esophageal cancer is one of the leading causes of cancer-related deaths worldwide. Its high invasiveness and delayed diagnosis result in poor patient outcomes. According to the World Health Organization, there were approximately 637,000 new cases and 516,000 deaths from esophageal cancer in 2022. The organization also clearly states that China accounts for 57.4% of global cases and 55.6% of deaths, representing an extremely heavy burden (1). Surgical resection is the primary treatment for patients with locally advanced esophageal cancer (T2-T4a, N0-N+). The choice of surgical technique directly impacts tumor resection completeness, perioperative safety, and long-term outcomes. However, optimizing surgical approaches—left thoracic approach (e.g., Sweet procedure) versus right thoracic approach (e.g., Ivor-Lewis or McKeown)—remains a contentious issue in thoracic surgery (2). High-quality, bias-mitigated medical evidence is necessary to guide clinical decision-making.

The left thoracic approach has long been considered the preferred method for treating lower esophageal cancer and tumors of the gastroesophageal junction due to its simplicity and minimal trauma from a single incision (3). However, it has anatomical limitations that result in inadequate dissection of the upper mediastinum and paraglossal lymph nodes, which may increase the risk of local recurrence (4). In contrast, the right thoracic approach provides more extensive exposure of the thoracic cavity and enhances thoracic lymph node dissection. This approach offers theoretical advantages in tumor control for mid-to-upper esophageal cancer (5). Nevertheless, the right thoracic approach is associated with a longer surgical duration and a higher risk of postoperative pulmonary complications, such as pneumonia and respiratory failure, raising concerns about its safety (6). In recent years, several observational studies and randomized controlled trials (RCTs) have attempted to compare the clinical outcomes of the two approaches, but the results are conflicting. Some studies support the survival benefits of the right thoracic approach (7). However, these findings are primarily derived from retrospective studies with inherent selection bias, which limits their ability to establish a definitive causal relationship. Other studies emphasize the advantages of the left thoracic approach in postoperative recovery (8). This controversy stems from the heterogeneity of the existing evidence (differences in surgical techniques and lymph node clearance standards) and the inadequate integration of long-term survival data.

Existing systematic reviews and meta-analyses often have limitations. For example, these analyses may focus on single outcome measures, such as perioperative mortality or the number of lymph nodes removed. They may also have small sample sizes and high methodological heterogeneity. Therefore, it is difficult to comprehensively assess the overall benefits of the two approaches (9). The meta-analysis by Xue et al. (10) restricted surgical sites to the middle and lower esophagus and surgical procedures to the Sweet procedure and Ivor-Lewis procedures. This restriction may cause the impact of lesion location on surgical strategies to be overlooked. Furthermore, the evidence may be outdated and insufficient to guide current surgical decision-making for esophageal cancer. Our meta-analysis overcomes the limitations of the middle-lower segment by covering the entire esophagus. It also includes the McKeown procedure for comparison, and updates and expands the range of outcome measures for greater comprehensiveness. Through a systematic search of the global literature and the use of evidence-based medicine methods, the following core indicators of right- and left-approach esophagectomy were quantitatively compared: i) perioperative safety (surgical duration, bleeding volume, and complication incidence); ii) oncological outcomes (number of lymph nodes removed and R0 resection rate); iii) long-term survival (overall survival [OS] and disease-free survival [DFS]). This analysis aims to provide surgeons with a scientific basis for selecting individualized surgical procedures and to promote the standardization and precision of esophagectomy by integrating high-quality data.

## 2 Methods

This meta-analysis adhered to the Preferred Reporting Items for Systematic Reviews and Meta-analyses guidelines (11). The protocol has been registered in the International Prospective Register of Systematic Reviews (CRD420251026319).

### 2.1 Search methods

The Cochrane Library, PubMed, Embase, and Web of Science were searched up to March 19, 2025. A combination of subject terms and free terms was used for the search. The Medical Subject Headings terms used were as follows: “Esophageal Squamous Cell Carcinoma”, “Esophagectomy”, “Cohort Studies”, “Observation”, and “Prospective Studies”. The specific search strategy is detailed in **Supplementary Table 1**. Additional searches were conducted in the reference lists of published reviews to ensure the most comprehensive literature retrieval possible.

### 2.2 Inclusion and exclusion criteria

The inclusion and exclusion criteria were based on the PICOS principles: population, intervention, comparison, outcome, and study design.

Inclusion: (i) **P**opulation: Patients diagnosed with esophageal cancer, regardless of age or disease severity; (ii) **I**ntervention: esophagectomy performed through a right thoracic approach; (iii) **C**omparison: esophagectomy performed through a left thoracic approach; (iv) **O**utcome: at least one surgery-related indicator, complication, or survival data. Primary outcomes included surgical duration and the number of lymph nodes removed during surgery. Secondary outcomes included postoperative hospital stay, anastomotic fistula, chylothorax, pulmonary complications, wound infection, OS rate, and DFS rate. (v) **S**tudy Design: cohort studies, RCTs, and non-randomized clinical trials comparing the two surgical approaches. Only publications in English were included.

Literature with the following characteristics was ruled out: (i) Animal or cell experiments, case reports, reviews, scientific experimental plans, letters, editorials, and conference papers. (ii) Missing or seriously erroneous research data. (iii) Full text not found.

### 2.3 Literature screening and data extraction

The retrieved articles were imported into EndNote X9 (Clarivate Analytics, London, England). Two researchers (Xiangdong Peng and Guixian Liu) independently reviewed the titles and abstracts, excluded irrelevant articles and duplicates, and read the full texts for a second screening. For the literature on which the two researchers disagreed, a re-evaluation was conducted after discussion with a third researcher. Using Excel 2016 (Microsoft, Redmond, Washington), two researchers independently extracted data from the final included literature, including the following: first author, publication year, country, study type, specific surgical types for the intervention and control groups, surgical duration, preoperative adjuvant therapy, tumor location, and outcome measures.

### 2.4 Quality assessment

Two researchers (Wen Liu and Banglin Xia) used three tools to appraise study quality: the Cochrane Risk of Bias Assessment Tool (RoB 2.0) (12), the Newcastle-Ottawa Scale (NOS) (13), and the Methodological Index for Non-Randomized Studies (MINORS) (14). The RoB 2.0 tool includes six domains: randomization, deviation from the intended intervention, missing outcome data, outcome measurement, selective reporting of results, and bias from other sources. For each study, each domain was assessed as “low risk”, “high risk”, or “possible risk”. For studies with disagreements, the assessment was made after discussion with a third researcher. The NOS primarily evaluates quality based on three areas and eight questions. Except for comparability, which is scored out of two points, the remaining seven questions are scored out of one point. Scores of seven to nine are considered high-quality research, while scores of four to seven are considered moderate-quality research. After the evaluation, two researchers cross-checked the results. If any discrepancies existed, a third researcher assisted in making a decision. The MINORS includes 12 evaluation items, each scored from zero to two points. A score of zero indicates no reporting; a score of one indicates reporting with incomplete information; and a score of two indicates reporting with sufficient information. The first eight items apply to studies without a control group and have a maximum score of 16 points. The additional four items apply to studies with a control group and have a maximum score of 24 points. After the assessment, two researchers cross-checked the results. If any discrepancies existed, a third researcher made the final determination.

### 2.5 Statistical analysis

Stata 15.0 was applied for the data analysis. Heterogeneity was quantified using Cochran’s Q test and Higgins I^2^. P < 0.10 or I^2^ > 50% denoted pronounced heterogeneity, and a random-effects model was adopted. Otherwise, a fixed-effects model was selected. When excessive heterogeneity was present, sensitivity analysis, subgroup analysis, and regression analysis were implemented to ascertain its sources. Funnel plots were utilized for visualizing publication bias, and the Egger’s test was employed for statistical testing of publication bias. When publication bias was present, the trim-and-fill method was used to evaluate its impact on the results of the meta-analysis. A p-value < 0.05 implied that the pooled statistics were statistically significant.

## 3 Results

**Figure 1:**
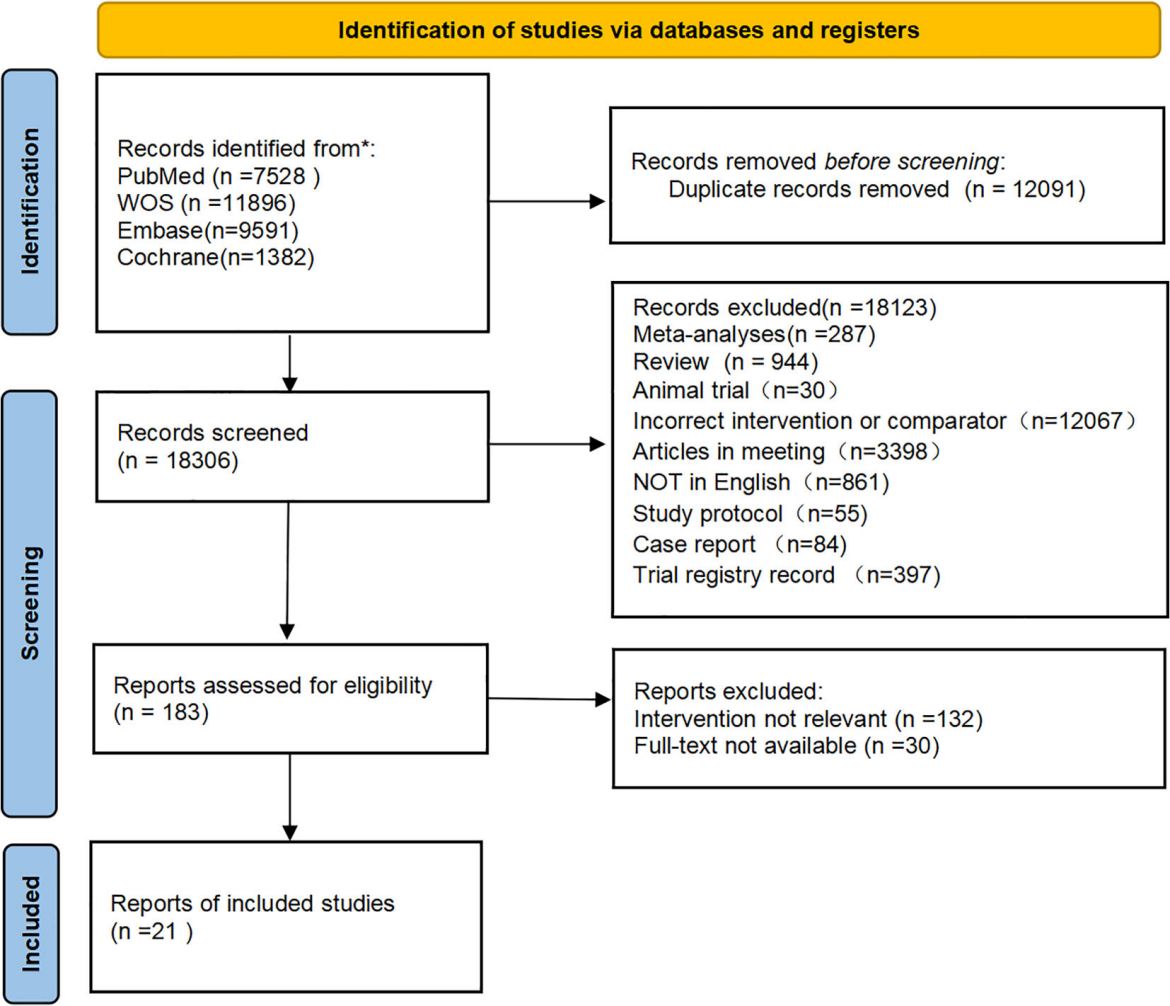
PRISMA flow chart.

A total of 30,397 articles were retrieved, from which 12,091 duplicates were excluded. After preliminary screening, an additional 12,423 articles were excluded. The remaining 183 articles were read in full. Finally, 21 articles (15–35) were included. The screening process is displayed in **Figure 1**.

### 3.1 Basic characteristics of included studies

The 21 included studies were from China and involved 17,195 patients with a mean age ranging from 55.66 to 73.32. The study types included three categories: RCT, cohort study, and non-RCT clinical trial. Details are listed in **Table 1**.

**Table 1:** Characteristics of the included studies.

No.	Study	Country	Research type (single/multicenter/database)	Study design	Sample size		Age (mean ± SD)		Sex (male/female)		The specific location of the tumor (number)		Specific surgical operations		Duration of surgery (Min)		Outcome measures	Quality evaluation score
					Intervention	Control	Intervention	Control	Intervention	Control	Intervention	Control	Intervention	Control	Intervention	Control		
1	Ma 2014 (15)	China	Single center	Retrospective cohort	167	748	>18	>18	141/26	608/140	Middle (111) Lower (56)	Middle(501) Lower(247)	Ivor- Lewis	Sweet	208 ± 63	181 ± 71	A,B,C,D,E,F,G	7*
2	Ma 2015 (17)	China	Database	Retrospective cohort	150	545	56.91 ± 8.42	55.66 ± 9.85	106/44	386/159	Upper (63) Middle(80) Lower (7)	Upper (15)Middle(405) Lower (125)	Right	Left	270.83 ± 68.38	189.24 ± 50.41	A,D,E,F,G,I	8*
3	Li 2015 (16)	China	Single center	RCT	150	150	59.66± 6.82	59.70 ± 6.63	118/32	124/26	Middle(95) Lower(55)	Middle(82) Lower(68)	Ivor- Lewis	Sweet	202 ± 38	174 ± 35	A, D, E, F	ROB2
4	Li 2025 (33)	China	Single center	RCT	146	140	60.91± 2.28	59.95 ± 1.72	114/32	116/24	Middle(92) Lower(54)	Middle(79) Lower(61)	Ivor- Lewis	Sweet	NR	NR	B,H,I	ROB2
5	Mu 2016 (18)	China	Single center	Retrospective cohort	45	1701	58.9 ± 7.6	59.9 ± 8.5	33/12	1408/299	Middle(35) Lower(10)	Middle (1652) Lower(49)	Ivor-Lewis	Sweet	390± 117	212 ± 48	A,B,D,E,G	7*
6	Liu 2017 (19)	China	Single center	Retrospective cohort	57	57	73.32± 1.74	72.82± 1.74	53/22	150/51	Upper(15) Middle(52) Lower(8)	Upper(4) Middle(123) Lower(74)	Right	Left	372± 120	192 ± 48	A,B,D,E,G	7*
7	Wang 2017 (20)	China	Single center	Retrospective cohort	M/I(546/204)	1535	>18	>18	NR	NR	NR	NR	McKeown/Ivor-Lewis	Sweet	NR	NR	D	6*
8	Wen 2017 (21)	China	Single center	Retrospective cohort	M/I( 26/47)	269	>18	>18	NR	NR	NR	NR	McKeown/Ivor-Lewis	Sweet	NR	NR	B, D,F,H	7*
9	Li 2018 (22)	China	Single center	Non-randomized controlled trial	21	28	60.1 ± 4.9	61.8 ± 5.1	19/2	28/0	Middle(14) Lower(4) Middle -lower(3)	Middle(13) Lower(13) Middle- Lower(2)	Right	Left	349.6 ± 86.3	363.4 ± 78.8	A,B,C,D,F,G	20^#^
10	Ding 2019 (23)	China	Single center	Retrospective cohort	232	143	NR	NR	163/69	111/32	Upper(4) Middle(138) Lower(90)	Upper(4) Middle(39) Lower(100)	Ivor- Lewis	Sweet	NR	NR	B,C,D,F	7*
11	Feng 2019 (24)	China	Single center	Retrospective cohort	75	75	60.49 ± 8.46	58.27± 8.32	62/13	55/20	NR	NR	Ivor- Lewis	Sweet	425.22 ± 74.05	293.58 ± 74.73	A,B,C,D	7*
12	Li 2019 (25)	China	Single center	Retrospective cohort	64	65	60 ± 2.57	59± 1.71	51/13	53/12	Middle(44) Lower(20)	Middle(36) Lower(29)	Right	Left	NR	NR	B,D,F,H,I	7*
13	Wang A 2019 (26)	China	Single center	Retrospective cohort	325	299	62.07 ± 1.56	62.05 ± 1.74	234/65	254/71	Middle(182) Lower(143)	Middle(181) Lower(118)	Ivor- Lewis	Sweet	165 ± 5.189	160.1317 ± 6.1093	A,B,C,D,E,F,H,I	8*
14	Wang B 2019 (27)	China	Database	Retrospective cohort	216	216	61.06 ± 2.17	61.46 ± 8.03	181/35	185/31	Upper and middle junction(11) Middle(99) Lower(106)	Upper and middle junction(10) Middle(100) Lower(106)	Ivor- Lewis	Sweet	265.9309 ± 21.0146	201.464 ± 10.145	A,B,C,D,E,F,H,I	7*
15	Chen 2020 (28)	China	Single center	Retrospective cohort	346	346	>18	>18	270/76	326/91	Upper (73) Middle (152) Lower (121)	Upper (17) Middle (143) Lower (186)	McKeown	Sweet	NR	NR	C,D,F,H,I	7*
16	Zheng 2020 (29)	China	The database of the department	Retrospective cohort	202	235	63.26 ± 6.21	64.75 ± 7.17	143/59	156/79	NR	NR	McKeown	Sweet	293.02 ± 314.94	201.77 ± 48.76	A,B,C,D,G,I	7*
17	Mao 2022 (30)	China	Multicenter	RCT	M/I(263/145)	453	60.6± 7.5	61.1± 6.9	341/67	369/84	Upper(20) Middle(266) Lower(122)	Upper(13) Middle(284) Lower(156)	McKeown/Ivor-Lewis	Sweet	274.48 ± 78.92	205.34 ± 51.47	A,B,C,D,E,F,G	ROB2
18	Yu 2022 (31)	China	Single center	Retrospective cohort	96	43	>18	>18	66/30	36/7	Middle(63) Distal(33)	Middle(29) Distal(14)	McKeown	Sweet	351 ± 36	282 ± 38	A,B,D,F	7*
19	Yang 2023 (34)	China	Single center	Retrospective cohort	532	453	61.98 ± 1.81	64.00 ± 1.67	381/151	312/141	Upper(136) Middle(317) Lower(79)	Upper(21) Middle(274) Lower(158)	Right	Left	NR	NR	H,I	8*
20	Liu 2024 (32)	China	Multicenter	Retrospective cohort	M/I (2123/97)	3336	>18	>18	1506/714	2129/1207	Upper(512) Middle(1399)Lower(309)	Upper(318) Middle(2255)Lower(763)	McKeown/Ivor-Lewis	Sweet	NR	NR	I	7*
21	Wang 2025 (35)	China	Single center	Retrospective cohort	61	22	61.2± 7.0	59.6± 7.8	54/7	19/3	Middle(29) Lower(32)	Middle(11) Lower(11)	McKeown/Ivor-Lewis	Sweet	356.5± 83.2	245.6 ± 27.8	A,B,C,D,F,G	8*

A: Surgical duration; B: Lymph nodes removed; C: Hospital stay; D: Anastomotic fistula; E: Wound infection; F: Chylothorax; G: Pulmonary complications; H: DFS (disease-free survival); I: OS (overall survival); *: Newcastle-Ottawa Scale score; #: Methodological Index for Non-randomized Studies Scale score; NR: Not reported; ROB2:**Supplementary Figure 1** Risk of bias assessment.

### 3.2 Results of the methodological quality assessment

The results of the risk of bias assessment are summarized in **Table 1**. Three RCTs (16, 30, 33) (**Supplementary Figure 1**) described the randomization process, had a low risk of missing data bias, and had no outcome measurement bias and no selective reporting bias. These studies had an overall low risk of bias. The NOS scores of the 17 cohort studies (15, 17–21, 23–29, 31, 32, 34, 35) were all between seven and eight points, indicating high quality. One non-RCT clinical trial (22) had a MINORS score of 20 points.

### 3.3 Analysis results

#### 3.3.1 Primary outcomes: intraoperative conditions

##### 3.3.1.1 Surgical duration

Thirteen articles (15–19, 22, 24, 26, 27, 29–31, 35) reported the surgical duration. Due to significant heterogeneity (I^2^ = 99.4%, P < 0.01), a random-effects model was adopted. The results showed a significantly longer surgical duration for the right approach compared to the left approach (mean difference [MD] = 77.51, 95% confidence interval [CI]: 53.19–101.84, P < 0.01) (**Supplementary Figure 2A**). Considering that four studies (18, 19, 24, 27) used propensity score matching and different surgical procedures, a subgroup analysis was conducted to determine if the heterogeneity in surgical duration could be reduced with well-matched data. The subgroup analysis of surgical duration showed significant differences in propensity score matching and surgical type ([matched: I^2^ = 97.4%; not matched: I^2^ = 98.6%; P < 0.05] [surgical type = I: I^2^ = 99.7%; surgical type = M: I^2^ = 0.0%; surgical type = right: I^2^ = 93.5%; P < 0.05) (**Supplementary Figure 3A**). The subgroup analysis data are detailed in **Table 2**. To determine if publication year and the number of lymph nodes removed significantly affected the difference in surgical duration, a further meta-regression analysis was conducted. The results revealed that neither publication year (P = 0.760) nor the number of lymph nodes removed (P = 0.595) could explain the heterogeneity in surgical duration between the right and left thoracic approaches.

**Table 2:** Subgroup analysis.

Outcome measures	Grouping factors		Studies	Effect size (95% CI)	I^2^	P
Surgical duration	Propensity score matching	Matched	4	137.21 (72.00-202.42)	97.4%	<0.0001
		Not Matched	9	52.29 (26.24-78.34)	98.6%	<0.0001
	Surgical type	I	6	68.85 (35.49-102.21)	99.7%	<0.0001
		M	2	70.91 (58.05-83.77)	0.0%	<0.0001
		Right	5	88.30 (56.76-119.83)	93.5%	<0.0001
Number of lymph nodes removed	Propensity score matching	Matched	4	6.27 (-0.89-13.42)	97.2%	0.089
		Not Matched	12	2.13 (0.31-3.94)	96.2%	0.022
	Surgical type	I	8	3.93 (-0.16-8.02)	99.2%	0.060
		M	3	1.13 (-3.13-5.40)	94.0%	0.602
		Right	6	2.66 (-1.27-6.60)	95.9%	0.185
Anastomotic fistula	Propensity score matching	Matched	5	2.03 (0.98-4.18)	65.0%	0.600
		Not Matched	15	2.08 (1.41-3.06)	63.9%	0.043
	Surgical type	I	9	1.51 (0.95-2.42)	50.5%	0.083
		M	5	2.89 (1.82-4.60)	54%	<0.0001
		Right	6	2.46 (1.06-5.67)	66.6%	0.035
Wound infection	Propensity score matching	Matched	3	1.18 (0.64-2.15)	0.0%	0.056
		Not Matched	5	2.03 (1.02-4.01)	64.5%	<0.0001
	Comorbidities	Not Report	6	1.72 (0.99-3.01)	64.4%	0.056
		Report	2	1.66 (0.40-6.81)	0.0%	0.483
DFS	Tumor location	Upper, Middle, Lower	4	1.00 (0.91, 1.10)	0.0%	0.996
		Middle, Lower	4	1.05 (0.69, 1.59)	83.1%	0.824
		Right, Unable to distinguish	1	0.95 (0.72, 1.25)	NA	0.715
OS	Tumor location	Upper, Middle, Lower	4	0.91 (0.85, 0.98)	28.2%	0.014
		Middle, Lower	4	1.15 (0.77, 1.74)	78.3%	0.496
		Right, Unable to distinguish	2	0.96 (0.79, 1.17)	0.0%	0.671

I, Ivor-Lewis; M, McKeown; NA, Not applicable.

##### 3.3.1.2 Number of lymph nodes removed

Sixteen studies (15, 17–19, 21–27, 29–31, 33, 35) reported the number of lymph nodes removed. Due to significant heterogeneity (I^2^ = 98.7%, P < 0.01), a random-effects model was used. The number of lymph nodes removed through the right approach was significantly higher than that through the left thoracic approach (MD = 3.00, 95% CI: 0.30–5.69, P = 0.029) (**Supplementary Figure 2B**). Considering four studies (18, 19, 24, 27) used propensity score matching and different surgical approaches, a subgroup analysis was conducted. Sixteen studies (15, 17–19, 21–27, 29–31, 33, 35) were included in the subgroup analysis involving 17 datasets. The analysis revealed significant differences in propensity score matching and surgical type ([matched: I^2^ = 97.2%; not matched: I^2^ = 96.2%] [surgical type = I: I^2^ = 99.2%; surgical type = M: I^2^ = 94%; surgical type = right: I^2^ = 95.9%) (**Supplementary Figure 3B**). Notably, considering that most clinical series harvested over 25 lymph nodes for adequate staging of esophageal cancer, the absolute magnitude of this difference (three additional nodes) was relatively small.

#### 3.3.2 Secondary outcome measures: postoperative outcomes (anastomotic fistula, wound infection, chylothorax, pulmonary complications, postoperative hospital stay)

Eighteen studies (15–31, 35) reported anastomotic fistulas postoperatively (**Supplementary Figure 2D**). Eight studies (15–19, 26, 27, 30) mentioned wound infections (**Supplementary Figure 2E**). Thirteen studies (15–17, 21–23, 26–28, 30, 31, 35) reported postoperative chylothorax (**Supplementary Figure 2F**). Eight studies (15, 17–19, 22, 29, 30, 35) mentioned postoperative pulmonary complications (**Supplementary Figure 3G**). Ten studies (15, 22–24, 26–30, 35) reported postoperative hospital stays (**Supplementary Figure 2C**). Due to significant heterogeneity in postoperative anastomotic fistulas (I^2^ = 63.2%, P < 0.01), wound infections (I² = 50.3%, P = 0.05), and hospital stays (I^2^ = 99.3%, P < 0.01), a random-effects model was adopted. The results showed that the risks of anastomotic fistula (RR = 2.07, 95% CI: 1.49-2.88, P < 0.01) and wound infection (risk ratio [RR] = 1.68, 95% CI: 1.04-2.73, P < 0.05) were higher with the right approach than with the left thoracic approach. However, no significant difference in postoperative hospital stay was observed between the two surgical approaches (MD = 1.02, 95% CI: -1.29–3.33, P = 0.38). Additionally, since there was no significant heterogeneity for chylothorax (I^2^ = 0.0%, P = 0.74) or pulmonary complications (I^2^ = 32%, P = 0.17), a fixed-effects model was chosen. The results showed no notable difference in the risk of chylothorax between the two surgical approaches (RR = 1.02, 95% CI: 0.72–1.45, P = 0.90). However, the risk of pulmonary complications was substantially higher with the right thoracic approach (RR = 1.39, 95% CI: 1.15–1.68, P < 0.01).

A subgroup analysis involving 20 datasets was conducted for anastomotic fistulas. The results revealed significant differences in propensity score matching and surgical type ([matched: I^2^ = 63.9%; not matched: I^2^ = 65.0%; P < 0.05] [surgical type = I: I^2^ = 50.5%; surgical type = M: I^2^ = 54.0%; surgical type = right: I^2^ = 66.6%]). McKeown (RR = 2.89, 95% CI: 1.82–4.60) may pose a higher risk of anastomotic fistula than Ivor-Lewis (RR: 1.51, 95% CI: 0.95–2.42).

To determine whether the number of lymph nodes removed affected the risk of anastomotic fistulas, a meta-regression analysis was conducted. Of the 18 studies (15–31, 35) examined, two (20, 28) that did not provide detailed information on intraoperative lymph node resection were excluded. After exclusion, the effect size decreased (RR = 2.07, 95% CI: 1.49–2.88 vs. RR = 1.76, 95% CI: 1.17–2.66), but the statistical significance remained unchanged (P < 0.0001 vs. P = 0.007). The meta-regression results, based on 16 studies (15–19, 21–27, 29–31, 35), revealed that the number of lymph nodes removed significantly affected the risk of anastomotic fistula (P < 0.01). A subgroup analysis of wound infection (matched: I^2^ = 0.0%; not matched: I^2^ = 64.5%; studies reporting patient comorbidities: I^2^ = 0.0%; studies not reporting comorbidities: I^2^ = 64.4%) revealed (**Supplementary Figure 3D**) that the risk of wound infection was significantly higher in the subgroup without propensity score matching for the right thoracic approach (RR = 2.03, 95% CI: 1.02–4.01). Additionally, the subgroup that did not report comorbidities indicated that the risk of wound infection from the right thoracic approach may be slightly higher than that from the left thoracic approach (RR = 1.72, 95% CI: 0.99–3.01).

#### 3.3.3 Survival outcomes

A meta-analysis of DFS was conducted based on eight studies (21, 25–28, 33–35) (**Supplementary Figure 2H**), which showed significant heterogeneity (I^2^ = 59.7%, P < 0.01). The random-effects model revealed no evidence of a difference in DFS between the right and left thoracic approaches (hazard ratio [HR] = 1.04, 95% CI: 0.90–1.19, P > 0.05). An analysis of OS was conducted based on ten studies (16, 26–30, 32–36) (**Supplementary Figure 2I**). Due to significant heterogeneity (I^2^ = 62.1%, P < 0.01), a random-effects model was selected. The comprehensive analysis revealed no evidence of a difference in OS between patients undergoing right and left thoracic approaches (HR = 0.98, 95% CI: 0.88–1.08, P > 0.05). The subgroup analyses of DFS (tumor location = upper, middle, lower, I^2^ = 0.0%, tumor location = middle, lower, I^2^ = 83.1%, tumor location = unable to distinguish, I^2^ = not applicable, P = 0.598) (**Supplementary Figure 3E**) and OS (tumor location = upper, middle, lower, I^2^ = 28.2%, tumor location = middle, lower, I^2^ = 78.3%, tumor location=unable to distinguish, I^2^ = 0.0%, P = 0.629) (**Supplementary Figure 3F**) showed no significant differences between the two groups.

#### 3.3.4 Sensitivity analysis

A sensitivity analysis was conducted to validate the stability of pooled results. The results across all outcomes remained robust. Details are presented in **Supplementary Figure 4**.

#### 3.3.5 Publication bias

An Egger’s test was performed for all previous analyses (**Table 3**). The results revealed no notable publication bias, except for surgical duration (P < 0.05). To correct for the observed publication bias in surgical duration, the trim-and-fill method was used (**Supplementary Figure 5**). This model identified six potential missing studies (left-sided), and the adjusted pooled effect size shifted from 77.51 (95% CI: 53.19–101.84) to 20.61 (95% CI: −1.02–42.24). This adjustment substantially altered the original estimate, resulting in a pooled effect that was no longer statistically significant.

**Table 3:** Egger’s test.

Outcome measures	t	p
Surgical duration	3.17	0.009
Lymph node dissection	-1.31	0.209
Hospital stay	0.25	0.809
Anastomotic fistula	-1.06	1.303
Wound infection	1.26	0.256
Chylothorax	0.81	0.435
Pulmonary complications	0.76	0.476
DFS	0.27	0.791
OS	0.41	0.692

## 4 Discussion

Esophageal cancer is a leading cause of cancer-related deaths worldwide. Delayed diagnosis and high invasiveness contribute to poor outcomes (36). While the field of esophageal surgery has recognized that removing more lymph nodes does not always significantly improve the 5-year OS (37, 38), removing a greater number of lymph nodes can significantly improve OS for patients with locally advanced disease (39, 40). This study found that the right thoracic approach was associated with a statistically significant increase in the number of lymph nodes removed compared with the left approach (P = 0.029). However, the absolute difference of three nodes is modest relative to the typical total lymph node yield (> 25 nodes) in clinical practice. This finding may reflect the superior thoracic exposure of the right approach, which facilitates access to lymph node stations in the upper mediastinum and paraglossal regions that are relatively difficult to reach through the left approach. However, the clinical significance of this numerical difference requires further validation in prospective studies, particularly with regard to its correlation with long-term oncological outcomes.

Our study showed that, compared with the left thoracic approach, the right thoracic approach significantly increased multiple perioperative risks. First, there was prolonged surgical duration (P < 0.01). The right thoracic approach often requires multiple incisions, such as the thoracoabdominal incision in the Ivor-Lewis procedure, and involves more complex mediastinal anatomy and more extensive lymph node dissection. Publication bias was detected for the outcome of surgical duration. After correcting for this bias using the trim-and-fill method, however, the difference in surgical duration between the two surgical procedures was no longer statistically significant. This finding revises the previous understanding of the time cost associated with these procedures. The initially observed significant increase in surgical duration with the right thoracic approach might have been exaggerated due to publication bias against small-sample studies with negative results. In practical clinical settings, this should be judged comprehensively by considering factors such as surgeon experience and hospital equipment. In addition, more large-scale, multicenter prospective studies are needed to clarify the true difference in surgical duration between the two approaches and reduce the influence of publication bias on clinical decision-making. Second, there is an increased risk of anastomotic fistula (P < 0.01). The incidence of anastomotic fistula was higher in the right thoracic approach (1.3%–26.3%) than in the left approach (1.8%–9%). Further meta-regression analysis revealed a positive correlation between anastomotic fistula risk and the number of lymph nodes removed (P < 0.01). This suggests that extensive lymph node dissection may impair the blood supply to the esophageal stump and gastrointestinal anastomosis, thereby increasing tissue injury. The risk of anastomotic fistula was significantly higher for the McKeown procedure than for the Ivor-Lewis procedure. The right thoracic approach exhibited the highest overall heterogeneity (I² = 66.6%). The McKeown procedure involves three incisions (cervical, thoracic, and abdominal) and a more extensive esophageal resection. The anastomosis is located in the neck, where the blood supply is relatively poorer. Intraoperative tissue ischemia-hypoxia and increased anastomotic tension are factors that collectively elevate the risk of fistula. Conversely, the Ivor-Lewis procedure involves two incisions (thoracic and abdominal), and the anastomosis is located below the aortic arch in the chest. The blood supply here is ensured by branches of the thoracic aorta. Its relatively simplified operative steps result in less traumatic stress that interferes with anastomotic healing. Additionally, the high I² value in the subgroup analysis suggests that confounders beyond the surgical procedure itself, such as patients’ baseline nutritional status, history of preoperative chemoradiotherapy, and surgeon experience, may influence the occurrence of fistulas. All of these factors should be considered in clinical practice. The continuously optimized anastomotic techniques (e.g., tube-stomach construction, mechanical anastomosis, and manual layered anastomosis) and perioperative management (e.g., nutritional support and monitoring) are crucial for reducing this risk. Third, there is an increased risk of wound infection (P < 0.05). Subgroup analysis revealed that this risk was more significant in studies that did not perform propensity score matching or report patient comorbidities. Finally, there is an increased risk of pulmonary complications (P < 0.01). From a technical perspective, the right thoracic approach usually requires one-lung ventilation. The confined operative space within the thoracic cavity and the direct traction on lung tissue can easily lead to pulmonary contusion and injury to the alveolar epithelial cells. This can contribute to postoperative pneumonia and atelectasis, which may exacerbate airway injury and increase the risk of pneumonia and respiratory failure.

This study provides important, evidence-based guidance on selecting surgical approaches for esophageal cancer. The surgical approach should balance tumor resection with perioperative risks, particularly for tumors in the upper thoracic esophagus. The left thoracic approach offers safety advantages, especially for tumors in the lower esophagus or gastroesophageal junction, for older patients, and for those with impaired cardiopulmonary function (41, 42). The right thoracic approach is more suitable for radical esophagectomy due to its advantages in clearing upper mediastinal lymph nodes, making it a better option for mid-to-upper esophageal cancer. The extensive experience of the surgical team is a key factor in performing complex approaches, such as the right thoracic approach (39, 40). Further study is needed to determine whether minimally invasive techniques (thoracoscopic/robot-assisted right thoracic approach) can reduce the risk of complications while maintaining oncological advantages.

Based on the highly heterogeneous existing evidence, our findings partially align with those of Xue et al. (10). For instance, the right thoracic approach yielded longer surgical durations and higher lymph node clearance rates than the left thoracic approach. The analysis of postoperative hospital stay and survival data revealed no significant differences between the two approaches. However, opinions differ. In our study, the risk of anastomotic fistula was significantly higher in the McKeown procedure than in the Ivor-Lewis procedure. This may be because the McKeown procedure employs a three-incision cervical anastomosis, where tension is higher, operative space is limited, and anastomotic technique demands greater precision. Furthermore, this surgical approach may increase pulmonary burden, potentially leading to more pulmonary complications, such as atelectasis and pneumothorax. Consequently, postoperative pulmonary complications were significantly elevated. This finding suggests that the high risk of anastomotic fistula associated with the right thoracic approach is not a universal characteristic but rather driven by the specific McKeown procedure. This discovery fills a gap in previous studies regarding the risk stratification of right thoracic approaches, making the comparative conclusions between right thoracic vs. left thoracic approaches more precise and clinically relevant (Clinically, the right thoracic approach often selects the McKeown or Ivor-Lewis procedure based on tumor location). Studies have shown (28, 43) that the incidence of cervical anastomotic fistula is higher than that of intrathoracic anastomosis, further validating the reliability of our research.

Postoperative complications (e.g., anastomotic fistula, pulmonary inflammation) are associated with the tumor immune microenvironment (TME) and host immune response. These complications influence long-term oncological outcomes. Liu et al. (44) identified a disulfidptosis signature that predicts the TME and prognosis in gastric cancer. Zhang et al. (45) proposed that combined detection of serum sTim-3, PG (especially the PG I/II ratio), and PD-L1 holds predictive value for the efficacy and prognosis of immune checkpoint inhibitor therapy in gastric cancer. High expression of sTim-3 and PD-L1 is associated with treatment failure and poor prognosis. Additionally, the HBV rtA181T mutation may be an indicator for assessing recurrence risk in upper gastrointestinal tumors by enhancing viral activity and inducing tumor suppressor gene defects (46). Due to their similar biological characteristics along the digestive tract, relevant research on gastric cancer (e.g., disulfidptosis-mediated TME regulation) can provide references for esophageal cancer. The expression and prognostic value of sTim-3, PG, and PD-L1 in esophageal cancer require further validation. Patients undergoing right transthoracic esophagectomy are at higher risk for complications. Preoperative detection of disulfidptosis-related biomarkers might predict the postoperative immune response and susceptibility to complications.

For early esophageal cancer and precancerous lesions, minimally invasive endoscopic interventions are a safer alternative to traditional thoracic surgery. Zheng et al. (47) demonstrated that multiband mucosectomy (MBM) and endoscopic submucosal dissection (ESD) both achieve satisfactory complete resection rates. However, MBM is associated with shorter hospital stays, faster recovery, and lower costs. ESD offers a higher en bloc resection rate. For eligible patients, choosing either technique over traditional surgery can reduce trauma and severe complications while achieving comparable oncological outcomes. This provides a translational strategy for lowering surgical complications in early esophageal cancer. For advanced cases requiring esophagectomy, pretreatment with endoscopy (e.g., partial lesion resection via ESD) might reduce tumor burden, though this requires further clinical validation.

Minimally invasive esophagectomy (MIE) has become the predominant approach due to reduced trauma and faster recovery. However, challenges remain regarding instrument precision and depth perception. Li et al. (48) proposed an artificial intelligence-driven framework that integrates instrument segmentation masks with RGB images to improve depth estimation accuracy in complex surgical scenarios. This technology could be applied to minimally invasive transthoracic esophagectomy to enhance spatial awareness and precision during surgery. For the right thoracic approach, which is associated with a longer surgical duration and a higher risk of complications, this technology could aid in anatomical identification, reduce inadvertent injury, shorten surgical duration, lower complication rates, and optimize the accuracy of lymph node dissection. This balances the oncological benefit against the risk of complications. Future work should explore clinical integration to improve the safety and efficacy of MIE.

Regarding survival outcomes, one of the key findings was that, although the right thoracic approach achieved a more thorough lymph node dissection, this anatomical advantage did not translate into significant survival benefits (OS: HR = 0.98, P > 0.05; DFS: HR = 1.04, P > 0.05). Subgroup analysis by tumor location (upper-middle segment, middle-lower segment, or undistinguishable) yielded consistent results. The potential reasons for this include: (i) the population included was predominantly East Asian, with a high prevalence of squamous cell carcinoma, whose lymph node metastasis patterns and sensitivity to clearance may differ from those of Western populations, which predominantly have adenocarcinoma; (ii) the widespread use of neoadjuvant/adjuvant therapy (e.g., chemoradiotherapy and immunotherapy) may have partially offset the impact of differences in surgical clearance range on prognosis (33); (iii) some studies had short follow-up periods, which may not be sufficient to capture long-term survival differences; and (iv) the biological behavior of the tumor itself (e.g., micrometastasis) may be a more critical prognostic factor. However, considering that these conclusions are drawn from a synthesis of low-quality retrospective evidence, further validation is required.

There are several limitations. First, our analysis included mainly observational studies (only three RCTs), which are subject to selection bias and confounding factors. Despite subgroup analyses (e.g., propensity score matching) and sensitivity analyses, residual confounding is inevitable. Second, there are significant differences among studies in terms of specific surgical techniques (e.g., Ivor-Lewis vs. McKeown), lymph node dissection criteria, anastomosis techniques, perioperative management, and neoadjuvant/adjuvant treatment regimens. These differences are the primary sources of result heterogeneity (e.g., surgical duration: I^2^ = 99.4%). Meanwhile, publication bias nullifies the difference in surgical duration. In the era of neoadjuvant therapy, the clinical significance of resecting three additional lymph nodes through the right thoracic approach requires further validation. These differences impact the comparability of results and the interpretation of pooled effect sizes. Third, most of the included studies were from China, and the patients were primarily diagnosed with esophageal squamous cell carcinoma. Conclusions for Western populations with a high incidence of esophageal adenocarcinoma should be interpreted with caution. Furthermore, since the present review did not search Chinese databases, future research should aim to incorporate and integrate Chinese-language literature to enhance comprehensiveness. Fourth, some of the included studies had short follow-up periods, which may have prevented the studies from adequately capturing long-term survival differences (e.g.,5-year OS). Additionally, including low-quality retrospective studies may have impacted the reliability of quality-of-life and other patient-reported outcomes. Ultimately, a regression analysis was not performed on the relationship between tumor location and survival outcomes because the original studies did not report tumor location and corresponding survival data.

More well-designed, multicenter, large-sample RCTs are needed to strictly standardize surgical procedures (Ivor-Lewis/McKeown and lymph node dissection range) and neoadjuvant/adjuvant treatment regimens. These trials must also ensure sufficient follow-up time (≥ 5 years) to accurately assess the long-term survival impact of different approaches on specific populations (e.g., different stages, locations, and pathological types). Additionally, we will explore whether thoracoscopy and robot-assisted right thoracic approaches can reduce surgical trauma and complications, especially pulmonary complications and anastomotic fistulas, while maintaining their oncological advantages in lymph node dissection. Furthermore, neoadjuvant therapy (e.g., chemotherapy, radiotherapy, and immunotherapy) is becoming the standard treatment. Therefore, it is essential to study the differences in efficacy between various surgical approaches within this model and develop a prognostic prediction model integrating clinical, pathological, and molecular markers.

## 5 Conclusion

This meta-analysis indicates that, compared with the left thoracic approach, the right-approach esophagectomy achieves more thorough lymph node dissection. However, it is also associated with a significantly increased risk of surgical duration, anastomotic fistula, wound infection, and pulmonary complications. The contradiction is that this anatomical advantage in dissection does not translate into benefits in OS or DFS. Therefore, clinical decisions must be individualized based on tumor location (tending toward the right chest in the middle and upper segments), the patient’s overall condition (cardiorespiratory function, age, and comorbidities), and the experience of a surgical team. The goal is to strike an optimal balance between achieving tumor eradication and ensuring perioperative safety. Future studies will require rigorous RCTs, exploration of minimally invasive techniques, and research conducted under neoadjuvant therapy settings to optimize strategies for selecting surgical approaches.

## References

[1] Bray F Laversanne M Sung H Ferlay J Siegel RL Soerjomataram I . Global cancer statistics 2022: GLOBOCAN estimates of incidence and mortality worldwide for 36 cancers in 185 countries. CA Cancer J Clin. (2024) 74:229–63. doi: 10.3322/caac.21834, PMID: 38572751

[2] Chai T Shen Z Chen S Lin Y Zhang Z Lin W . Right versus left thoracic approach oesophagectomy for oesophageal cancer: a systematic review and meta-analysis protocol. BMJ Open. (2019) 9:e030157. doi: 10.1136/bmjopen-2019-030157, PMID: 31289096 PMC6629400

[3] Guo Q Huang B Zhao J Ma Y Yuan D Yang Y . Perioperative pharmacological thromboprophylaxis in patients with cancer: A systematic review and meta-analysis. Ann Surg. (2017) 265:1087–93. doi: 10.1097/SLA.0000000000002074, PMID: 27849664

[4] Li H Chen S Cheng L Guo Y Lai H Li Y . Chinese guideline on sublingual immunotherapy for allergic rhinitis and asthma. J Thorac Dis. (2019) 11:4936–50. doi: 10.21037/jtd.2019.12.37, PMID: 32030209 PMC6988076

[5] van der Sluis PC Tagkalos E Hadzijusufovic E Babic B Uzun E van Hillegersberg R . Robot-assisted minimally invasive esophagectomy with intrathoracic anastomosis (Ivor lewis): promising results in 100 consecutive patients (the european experience). J Gastrointest Surg. (2021) 25:1–8. doi: 10.1007/s11605-019-04510-8, PMID: 32072382 PMC7850999

[6] Wang H Tang H Fang Y Tan L Yin J Shen Y . Morbidity and mortality of patients who underwent minimally invasive esophagectomy after neoadjuvant chemoradiotherapy vs neoadjuvant chemotherapy for locally advanced esophageal squamous cell carcinoma: A randomized clinical trial. JAMA Surg. (2021) 156:444–51. doi: 10.1001/jamasurg.2021.0133, PMID: 33729467 PMC7970392

[7] Schmoll HJ . Selective internal radiotherapy in advanced colorectal cancer: only for right-sided tumours? Lancet Oncol. (2017) 18:1138–9. doi: 10.1016/S1470-2045(17)30589-2, PMID: 28781172

[8] Liu Z Yang R Sun Y . Tubeless uniportal thoracoscopic wedge resection with modified air leak test and chest tube drainage. BMC Surg. (2020) 20:301. doi: 10.1186/s12893-020-00910-9, PMID: 33256711 PMC7706205

[9] Hewitt DB Brown ZJ Pawlik TM . Surgical management of intrahepatic cholangiocarcinoma. Expert Rev Anticancer Ther. (2022) 22:27–38. doi: 10.1080/14737140.2022.1999809, PMID: 34730474

[10] Xue Y Chen D Wang W Wang W Chen L Sang Y . Comparison of Ivor Lewis and Sweet esophagectomy for middle and lower esophageal squamous cell carcinoma: A systematic review and pooled analysis. EClinicalMedicine. (2020) 27:100497. doi: 10.1016/j.eclinm.2020.100497, PMID: 33089129 PMC7559873

[11] Moher D Liberati A Tetzlaff J Altman DG . Preferred reporting items for systematic reviews and meta-analyses: the PRISMA statement. Bmj. (2009) 339:b2535. doi: 10.1136/bmj.b2535, PMID: 19622551 PMC2714657

[12] Higgins JPT TJ Chandler J Cumpston M Li T Page MJ Welch VA . Cochrane Handbook for Systematic Reviews of Interventions (version 6.3) ( London, UK Cochrane Collaboration) (2022).

[13] Wells GA Wells G Shea B Shea B O’Connell D Peterson J . The Newcastle-Ottawa Scale (NOS) for Assessing the Quality of Nonrandomised Studies in Meta-Analyses. Newcastle, Ottawa: University of Newcastle/ Ottawa Hospital Research Institute (2014).

[14] Slim K Nini E Forestier D Kwiatkowski F Panis Y Chipponi J . Methodological index for non-randomized studies (minors): development and validation of a new instrument. ANZ J Surg. (2003) 73:712–6. doi: 10.1046/j.1445-2197.2003.02748.x, PMID: 12956787

[15] Ma J Zhan C Wang L Jiang W Zhang Y Shi Y . The sweet approach is still worthwhile in modern esophagectomy. Ann Thorac Surg. (2014) 97:1728–33. doi: 10.1016/j.athoracsur.2014.01.034, PMID: 24650587

[16] Li B Xiang J Zhang Y Li H Zhang J Sun Y . Comparison of Ivor-Lewis vs Sweet esophagectomy for esophageal squamous cell carcinoma: a randomized clinical trial. JAMA Surg. (2015) 150:292–8. doi: 10.1001/jamasurg.2014.2877, PMID: 25650816

[17] Ma Q Liu W Long H Rong T Zhang L Lin Y . Right versus left transthoracic approach for lymph node-negative esophageal squamous cell carcinoma. J Cardiothorac Surg. (2015) 10:123. doi: 10.1186/s13019-015-0328-4, PMID: 26384482 PMC4575477

[18] Mu JW Gao SG Xue Q Mao YS Wang DL Zhao J . The impact of operative approaches on outcomes of middle and lower third esophageal squamous cell carcinoma. J Thorac Dis. (2016) 8:3588–95. doi: 10.21037/jtd.2016.12.42, PMID: 28149553 PMC5227276

[19] Liu Q Chen J Wen J Yang H Hu Y Luo K . Comparison of right- and left-approach esophagectomy for elderly patients with operable thoracic esophageal squamous cell carcinoma: a propensity matched study. J Thorac Dis. (2017) 9:1883–90. doi: 10.21037/jtd.2017.06.22, PMID: 28839986 PMC5543008

[20] Wang W Zhao G Wu L Dong Y Zhang C Sun L . Risk factors for anastomotic leakage following esophagectomy: Impact of thoracic epidural analgesia. J Surg Oncol. (2017) 116:164–71. doi: 10.1002/jso.24621, PMID: 28384375

[21] Shiwang Wen LH Zhang Y Tian Z Li Y Lv H Xu Y . Comparison of survival rate, complications and life quality after different surgical procedures in esophageal cancer. Int J Clin Exp Pathology. (2017) 10:1886–97.

[22] Li X Wang W Zhou Y Yang D Wu J Zhang B . Efficacy comparison of transcervical video-assisted mediastinoscopic lymphadenectomy combined with left transthoracic esophagectomy versus right transthoracic esophagectomy for esophageal cancer treatment. World J Surg Oncol. (2018) 16:25. doi: 10.1186/s12957-017-1268-3, PMID: 29426329 PMC5807757

[23] Ding Q Zhou W Xue Y Han X Yin D Xue L . Comparison of postoperative complications between different operation methods for esophageal cancer. Thorac Cancer. (2019) 10:1669–72. doi: 10.1111/1759-7714.13092, PMID: 31245903 PMC6669799

[24] Feng Y Wu N Yan S Wang X Yang Y . Comparison of Ivor Lewis esophagectomy and Sweet esophagectomy for the treatment of middle-lower esophageal squamous cell carcinoma. J Thorac Dis. (2019) 11:3584–92. doi: 10.21037/jtd.2019.07.68, PMID: 31559065 PMC6753411

[25] Li B Hu H Zhang Y Zhang J Sun Y Xiang J . Esophageal squamous cell carcinoma patients with positive lymph nodes benefit from extended radical lymphadenectomy. J Thorac Cardiovasc Surg. (2019) 157:1275–83.e1. doi: 10.1016/j.jtcvs.2018.11.094, PMID: 33198003

[26] Wang J Wei N Jiang N Lu Y Zhang X . Comparison of Ivor-Lewis versus Sweet procedure for middle and lower thoracic esophageal squamous cell carcinoma: A STROBE compliant study. Med (Baltimore). (2019) 98:e14416. doi: 10.1097/MD.0000000000014416, PMID: 30732195 PMC6380795

[27] Wang Q Wu Z Zhan T Fang S Zhang S Shen G . Correction to: Comparison of minimally invasive Ivor Lewis esophagectomy and left transthoracic esophagectomy in esophageal squamous cell carcinoma patients: a propensity score-matched analysis. BMC Cancer. (2020) 20:593. doi: 10.1186/s12885-020-06999-8, PMID: 32586288 PMC7315537

[28] Chen D Hu Y Chen Y Hu J Wen Z . Comparison of outcomes between mcKeown and sweet esophagectomy in the elderly patients for esophageal squamous cell carcinoma: A propensity score-matched analysis. Cancer Control. (2020) 27:1073274820904700. doi: 10.1177/1073274820904700, PMID: 32048521 PMC7020469

[29] Zheng Y Li Y Liu X Zhang R Sun H Xing W . Right compared with left thoracic approach esophagectomy for patients with middle esophageal squamous cell carcinoma. Front Oncol. (2020) 10:536842. doi: 10.3389/fonc.2020.536842, PMID: 33194596 PMC7649421

[30] Mao YS Gao SG Li Y Hao AL Liu JF Li XF . Efficacy and safety of esophagectomy via left thoracic approach versus via right thoracic approach for middle and lower thoracic esophageal cancer: a multicenter randomized clinical trial (NST1501). Ann Transl Med. (2022) 10:904. doi: 10.21037/atm-22-3810, PMID: 36111056 PMC9469177

[31] Yu F Zhang Y Xu H Li K Gheng J Lin C . Comparison of McKeown Minimally Invasive Esophagectomy vs sweet esophagectomy for esophageal squamous cell carcinoma: A retrospective study. Front Oncol. (2022) 12:1009315. doi: 10.3389/fonc.2022.1009315, PMID: 36601481 PMC9806205

[32] Liu F Yang W He Y Yang W Chen L Xu R . Surgical quality determines the long-term survival superiority of right over left thoracic esophagectomy for localized esophageal squamous cell carcinoma patients: a real-world multicenter study. Int J Surg. (2024) 110:675–83. doi: 10.1097/JS9.0000000000000897, PMID: 37983771 PMC10871567

[33] Li B Wang Z Sun Y Hu H Zhang Y Xiang J . Ten-year survivals of right thoracic vs left thoracic approach for esophageal cancer. Ann Thorac Surg. (2025) 119:643–50. doi: 10.1016/j.athoracsur.2024.09.006, PMID: 39293749

[34] Yang Y Xin X Chen P Shi X Yang C Fan J . Left compared with right thoracic approach thoracotomy in esophageal cancer: a retrospective cohort study. J Cancer Res Clin Oncol. (2023) 149:8289–96. doi: 10.1007/s00432-023-04765-4, PMID: 37071207 PMC11797362

[35] Wang K Xie X He J Fang S Zhong Y Wu D . Right versus left thoracic approach esophagectomy for patients with neoadjuvant immunochemotherapy. Ann Med. (2025) 57:2456691. doi: 10.1080/07853890.2025.2456691, PMID: 39862207 PMC11770869

[36] Sheikh M Roshandel G McCormack V Malekzadeh R . Current status and future prospects for esophageal cancer. Cancers (Basel). (2023) 15:765. doi: 10.3390/cancers15030765, PMID: 36765722 PMC9913274

[37] Lagergren J Mattsson F Zylstra J Chang F Gossage J Mason R . Extent of lymphadenectomy and prognosis after esophageal cancer surgery. JAMA Surg. (2016) 151:32–9. doi: 10.1001/jamasurg.2015.2611, PMID: 26331431

[38] van der Schaaf M Johar A Wijnhoven B Lagergren P Lagergren J . Extent of lymph node removal during esophageal cancer surgery and survival. J Natl Cancer Inst. (2015) 107. doi: 10.1093/jnci/djv043, PMID: 25748792

[39] Wu Y Gu W Du B Lv C Yao N Zhu Y . Impact of the number of lymph node dissections and a novel risk stratification on the prognosis in elderly locally advanced esophageal adenocarcinoma. J Cancer. (2024) 15:4197–204. doi: 10.7150/jca.96574, PMID: 38947388 PMC11212079

[40] Li K Leng X He W Du K Li C Liu K . Resected lymph nodes and survival of patients with esophageal squamous cell carcinoma: an observational study. Int J Surg. (2023) 109:2001–9. doi: 10.1097/JS9.0000000000000436, PMID: 37222685 PMC10389544

[41] Matsuda S Takeuchi H Kawakubo H Kitagawa Y . Three-field lymph node dissection in esophageal cancer surgery. J Thorac Dis. (2017) 9:S731–s40. doi: 10.21037/jtd.2017.03.171, PMID: 28815069 PMC5538994

[42] Matsuda S Takeuchi M Kawakubo H Kitagawa Y . Lymph node metastatic patterns and the development of multidisciplinary treatment for esophageal cancer. Dis Esophagus. (2023) 36:doad006. doi: 10.1093/dote/doad006, PMID: 36857594 PMC10061432

[43] Hua F Sun D Zhao X Song X Yang W . Update on therapeutic strategy for esophageal anastomotic leak: A systematic literature review. Thorac Cancer. (2023) 14:339–47. doi: 10.1111/1759-7714.14734, PMID: 36524684 PMC9891862

[44] Liu Z Sun L Zhu W Zhu J Wu C Peng X . Disulfidptosis signature predicts immune microenvironment and prognosis of gastric cancer. Biol Direct. (2024) 19:65. doi: 10.1186/s13062-024-00518-6, PMID: 39148138 PMC11325698

[45] Zhang H Jiang Y Luo J Tan Q Xu M He B . Predictive and prognostic value of combined detection of sTim-3, PG and PD-L1 in immune checkpoint inhibitor therapy for advanced gastric cancer. Am J Transl Res. (2024) 16:6955–63. doi: 10.62347/MJOA5699, PMID: 39678619 PMC11645554

[46] Ning Q Yang T Guo X Huang Y Gao Y Liu M . CHB patients with rtA181T-mutated HBV infection are associated with higher risk hepatocellular carcinoma due to increases in mutation rates of tumour suppressor genes. J Viral Hepat. (2023) 30:951–8. doi: 10.1111/jvh.13886, PMID: 37735836

[47] Zheng J Yang J Zhao Z . Comparison of MBM and ESD in the treatment of single early esophageal cancer and precancerous lesions. Ann Ital Chir. (2024) 95:534–41. doi: 10.62713/aic.3416, PMID: 39186346

[48] Li X Chen W Duan X Gu X Li C . Collaborative surgical instrument segmentation for monocular depth estimation in minimally invasive surgery. Med Image Anal. (2025) 106:103765. doi: 10.1016/j.media.2025.103765, PMID: 40848507

