# Supplementary material for: Comparison of right- and left-approach esophagectomy for esophageal cancer: a meta-analysis
Source: Front Oncol. 2026 Jan 23;15:1685103. doi: 10.3389/fonc.2025.1685103 (PMC12875970; doi:10.3389/fonc.2025.1685103)
Supplement: Supplementary file 1 [file DataSheet1.docx]

**Supplementary Materials**

**Supplementary Table 1 Search Strategies**

**1) Pubmed search strategy**

| 1. Esophageal Squamous Cell Carcinoma [Mesh] |
| --- |
| 1. Esophageal carcinoma [Title/Abstract] |
| 1. ESCC [Title/Abstract] |
| 1. Esophageal Neoplasms [Mesh] |
| 1. Esophageal Neoplasm* [Title/Abstract] |
| 1. Esophagus Neoplasm [Title/Abstract] |
| 1. Esophagus Neoplasm*[Title/Abstract] |
| 1. Cancer of Esophagus [Title/Abstract] |
| 1. Esophageal Cancer* [Title/Abstract] |
| 1. Cancer of the Esophagus [Title/Abstract] |
| 1. Esophagus Cancer* [Title/Abstract] |
| 1. 1 OR 2 OR 3 OR 4 OR 5 OR 6 OR 7 OR 8 OR 9 OR 10 OR 11 |
| 1. Esophagectomy[Mesh] |
| 1. Esophagectomies [Title/Abstract] |
| 1. Carcinoma, Ivor Lewis [Title/Abstract] |
| 1. Carcinoma, Sweet [Title/Abstract] |
| 1. Carcinoma, McKeown [Title/Abstract] |
| 1. Ivor Lewis [Title/Abstract] |
| 1. Sweet [Title/Abstract] |
| 1. McKeown [Title/Abstract] |
| 1. Esophagus, Ivor Lewis [Title/Abstract] |
| 1. Esophagus, Sweet [Title/Abstract] |
| 1. Esophagus,McKeown [Title/Abstract] |
| 1. Esophageal, Ivor Lewis[Title/Abstract] |
| 1. Esophageal, Sweet [Title/Abstract] |
| 1. Esophageal, McKeown[Title/Abstract] |
| 1. Esophageal Squamous Cell Carcinoma, left, right[Title/Abstract] |
| 1. Esophageal Carcinoma, left, right[Title/Abstract] |
| 1. Ivor Lewis, Sweet[Title/Abstract] |
| 1. Esophagectomy, Ivor Lewis[Title/Abstract] |
| 1. Esophagectomy, Sweet[Title/Abstract] |
| 1. Esophagectomy,McKeown[Title/Abstract] |
| 1. Esophagectomy, left, right[Title/Abstract] |
| 1. 13 OR 14 OR 15 OR 16 OR 17 OR 18 OR 19 OR 20 OR 21 OR 22 OR 23 OR 24 OR 25 OR 26 OR 27 OR 28 OR 29 OR 30 OR 31 OR 32 OR33 |
| 1. Cohort Studies[MeSH Terms] |
| 1. Observation[MeSH Terms] |
| 1. Prospective Studies[MeSH Terms] |
| 1. Longitudinal Studies[MeSH Terms] |
| 1. Observational Study[Publication Type] |
| 1. Follow-Up Studies[MeSH Terms] |
| 1. Case-Control Studies[MeSH Terms] |
| 1. Cohorts[Title/Abstract] |
| 1. Cohort[Title/Abstract] |
| 1. Cohort Studies[Title/Abstract] |
| 1. Concurrent Study[Title/Abstract] |
| 1. Incidence Studies[Title/Abstract] |
| 1. Observation[Title/Abstract] |
| 1. Prospective Studies[Title/Abstract] |
| 1. Prospectively[Title/Abstract] |
| 1. Prospective[Title/Abstract] |
| 1. Longitudinal[Title/Abstract] |
| 1. Observational[Title/Abstract] |
| 1. Follow-Up[Title/Abstract] |
| 1. Nested[Title/Abstract] |
| 1. Relative risk[Title/Abstract] |
| 1. Hazard ratio[Title/Abstract] |
| 1. RR[Title/Abstract] |
| 1. HR[Title/Abstract] |
| 1. RCT[MeSH Terms] |
| 1. Randomized controlled trial[Title/Abstract] |
| 1. Controlled clinical trial[Title/Abstract] |
| 1. Random*[Title/Abstract] |
| 1. Placebo*[Title/Abstract] |
| 1. Group*[Title/Abstract] |
| 1. Control*[Title/Abstract] |
| 1. 35 OR 36 OR 37 OR 38 OR 39 OR 40 OR 41 OR 42 OR 43 OR 44 OR 45 OR 46 OR 47 OR 48 OR 49 OR 50 51 OR 52 OR 53 OR 54 OR 55 OR 56 OR 57 OR 58 OR 59 OR 60 OR 61 OR 62 OR 63 OR 64 OR 65 |
| 1. 12 AND 34 AND 66 |

**2) Embase search strategy**

| 1. ' Esophageal squamous cell carcinoma'/exp |
| --- |
| 1. ' Esophageal squamous cell carcinoma ':ti,ab,kw |
| 1. ' Esophageal carcinoma ':ti,ab,kw |
| 1. ' Escc':ti,ab,kw |
| 1. ' Esophageal neoplasms ': ti,ab,kw |
| 1. ' Esophageal neoplasm* ': ti,ab,kw |
| 1. ' Esophagus neoplasm ': ti,ab,kw |
| 1. ' Esophagus neoplasm* ':ti,ab,kw |
| 1. ' Cancer of esophagus ':ti,ab,kw |
| 1. ' Esophageal cancer* ':ti,ab,kw |
| 1. ' Cancer of the esophagus ':ti,ab,kw |
| 1. ' Esophagus cancer* ':ti,ab,kw |
| 1. 1 OR 2 OR 3 OR 4 OR 5 OR 6 OR 7 OR 8 OR 9 OR 10 OR 11 OR 12 |
| 1. ' Esophagectomy '/exp |
| 1. ' Esophagectomy ':ti,ab,kw |
| 1. ' Esophagectomies ':ti,ab,kw |
| 1. ' Carcinoma, ivor lewis ':ti,ab,kw |
| 1. ' Carcinoma, sweet ':ti,ab,kw |
| 1. ' Carcinoma, mckeown ':ti,ab,kw |
| 1. ' Ivor lewis ':ti,ab,kw |
| 1. ' Sweet ':ti,ab,kw |
| 1. ' Mckeown ':ti,ab,kw |
| 1. ' Esophagus, ivor lewis ':ti,ab,kw |
| 1. ' Esophagus, sweet ':ti,ab,kw |
| 1. ' Esophagus,mckeown ':ti,ab,kw |
| 1. ' Esophageal, ivor lewis ':ti,ab,kw |
| 1. ' Esophageal, sweet ':ti,ab,kw |
| 1. ' Esophageal, mckeown ':ti,ab,kw |
| 1. ' Esophageal squamous cell carcinoma, left, right ':ti,ab,kw |
| 1. ' Esophageal carcinoma, left, right ':ti,ab,kw |
| 1. ' Ivor-lewis, sweet ':ti,ab,kw |
| 1. ' Esophagectomy, ivor ':ti,ab,kw |
| 1. ' Esophagectomy, sweet ':ti,ab,kw |
| 1. ' Esophagectomy,mckeown ':ti,ab,kw |
| 1. ' Esophagectomy, left, right ':ti,ab,kw |
| 1. 14 OR 15 OR 16 OR 17 OR 18 OR 19 OR 20 OR 21 OR 22 OR 23 OR 24 OR 25 OR 26 OR 27 OR 28 OR 29 OR 30 OR 31 OR 32 OR 33 OR 34 OR 35 |
| 1. ' Cohort studies '/exp |
| 1. ' Cohort studies ':ti,ab,kw |
| 1. ' Longitudinal studies ':ti,ab,kw |
| 1. ' Case-control studies ':ti,ab,kw |
| 1. ' Follow-up studies ':ti,ab,kw |
| 1. ' Cohorts ':ti,ab,kw |
| 1. ' Cohort ':ti,ab,kw |
| 1. ' Cohort studies ':ti,ab,kw |
| 1. ' Concurrent study ':ti,ab,kw |
| 1. ' Incidence studies ':ti,ab,kw |
| 1. ' Observation ':ti,ab,kw |
| 1. ' Prospective studies ':ti,ab,kw |
| 1. ' Prospectively ':ti,ab,kw |
| 1. ' Prospective ':ti,ab,kw |
| 1. ' Longitudinal ':ti,ab,kw |
| 1. ' Observational ':ti,ab,kw |
| 1. ' Follow-up ':ti,ab,kw |
| 1. ' Nested ':ti,ab,kw |
| 1. ' Relative risk ':ti,ab,kw |
| 1. ' Hazard ratio ':ti,ab,kw |
| 1. ' RR ':ti,ab,kw |
| 1. ' HR':ti,ab,kw |
| 1. ' Observational study ':ti,ab,kw |
| 1. ' Rct ':ti,ab,kw |
| 1. ' Randomized controlled trial ':ti,ab,kw |
| 1. ' Controlled clinical trial ':ti,ab,kw |
| 1. ' Random* ':ti,ab,kw |
| 1. ' Placebo*t ':ti,ab,kw |
| 1. ' Group* ':ti,ab,kw |
| 1. ' Control* ':ti,ab,kw |
| 1. 37 OR 38 OR 39 OR 40 OR 41 OR 42 OR 43 OR 44 OR 45 OR 46 OR 47 OR 48 OR 49 OR 50 OR 51 OR 52 OR 53 OR 54 OR 55 OR 56 OR 57 OR 58 OR 59 OR 60 OR 61 OR 62 OR 63 OR 64 OR 65 OR 66 |
| 1. 13 AND 36 AND 67 |

**3) Web of science search strategy**

| 1. Esophageal Squamous Cell Carcinoma Subject search |
| --- |
| 1. Esophageal carcinoma Subject search |
| 1. ESCC Subject search |
| 1. Esophageal Neoplasms Subject search |
| 1. Esophageal Neoplasm* Subject search |
| 1. Esophagus Neoplasm Subject search |
| 1. Esophagus Neoplasm* Subject search |
| 1. Cancer of Esophagus Subject search |
| 1. Esophageal Cancer* Subject search |
| 1. Cancer of the Esophagus Subject search |
| 1. Esophagus Cancer* Subject search |
| 1. 12 1 OR 2 OR 3 OR 4 OR 5 OR 6 OR 7 OR 8 OR 9 OR 10 OR 11 |
| 1. Esophagectomy Subject search |
| 1. Esophagectomies Subject search |
| 1. Carcinoma, Ivor Lewis Subject search |
| 1. Carcinoma, Sweet Subject search |
| 1. Carcinoma, McKeown Subject search |
| 1. Ivor Lewis Subject search |
| 1. Sweet Subject search |
| 1. McKeown Subject search |
| 1. Esophagus, Ivor Lewis Subject search |
| 1. Esophagus, Sweet Subject search |
| 1. Esophagus,McKeown Subject search |
| 1. Esophageal, Ivor Lewis Subject search |
| 1. Esophageal, Sweet Subject search |
| 1. Esophageal, McKeown Subject search |
| 1. Esophageal Squamous Cell Carcinoma, left, right Subject search |
| 1. Esophageal Carcinoma, left, right Subject search |
| 1. Ivor-Lewis, Sweet Subject search |
| 1. Esophagectomy, Ivor Subject search |
| 1. Esophagectomy, Sweet Subject search |
| 1. Esophagectomy,McKeown Subject search |
| 1. Esophagectomy, left, right Subject search |
| 1. 13 OR 14 OR 15 OR 16 OR 17 OR 18 OR 19 OR 20 OR 21 OR 22 OR 23 OR 24 OR 25 OR 26 OR 27 OR 28 OR 29 OR 30 OR 31 OR 32 OR 33 |
| 1. Cohort Studies Subject search |
| 1. Observation Subject search |
| 1. Prospective Studies Subject search |
| 1. Longitudinal Studies Subject search |
| 1. Observational Study Subject search |
| 1. Follow-Up Studies Subject search |
| 1. Case-Control Studies Subject search |
| 1. Cohorts Subject search |
| 1. Cohort Subject search |
| 1. Cohort Studies Subject search |
| 1. Concurrent Study Subject search |
| 1. Incidence Studies Subject search |
| 1. Observation Subject search |
| 1. Prospective Studies Subject search |
| 1. Prospectively Subject search |
| 1. Prospective Subject search |
| 1. Longitudinal Subject search |
| 1. Observational Subject search |
| 1. Follow-Up Subject search |
| 1. Nested Subject search |
| 1. Relative risk Subject search |
| 1. Hazard ratio Subject search |
| 1. RR Subject search |
| 1. HR Subject search |
| 1. RCT Subject search |
| 1. Randomized controlled trial Subject search |
| 1. Controlled clinical trial Subject search |
| 1. Random* Subject search |
| 1. Placebo* Subject search |
| 1. Group* Subject search |
| 1. Control* Subject search |
| 1. 35 OR 36 OR 37 OR 38 OR 39 OR 40 OR 41 OR 42 OR 43 OR 44 OR 45 OR 46 OR 47 OR 48 OR 49 OR 50 OR 51 OR 52 OR 53 OR 54 OR 55 OR 56 OR 57 OR 58 OR 59 OR 60 OR 61 OR 62 OR 63 OR 64 OR 65 OR 66 |
| 1. 12 AND 34 AND 66 |

1. **Cochrane Library search strategy**

| 1. Esophageal Squamous Cell Carcinoma:ti,ab,kw |
| --- |
| 1. Esophageal carcinoma: ti,ab,kw |
| 1. ESCC:ti,ab,kw |
| 1. Esophageal Neoplasms:ti,ab,kw |
| 1. Esophageal Neoplasm*：ti,ab,kw |
| 1. Esophagus Neoplasm：ti,ab,kw |
| 1. Esophagus Neoplasm*：ti,ab,kw |
| 1. Cancer of Esophagus：ti,ab,kw |
| 1. Esophageal Cancer*：ti,ab,kw |
| 1. Cancer of the Esophagus：ti,ab,kw |
| 1. Esophagus Cancer*：ti,ab,kw |
| 1. 1 OR 2 OR 3 OR 4 OR 5 OR 6 OR 7 OR 8 OR 9 OR 10 OR 11 |
| 1. Esophagectomy: ti,ab,kw |
| 1. Esophagectomies: ti,ab,kw |
| 1. Carcinoma, Ivor Lewis: ti,ab,kw |
| 1. Carcinoma, Sweet: ti,ab,kw |
| 1. Carcinoma, McKeown: ti,ab,kw |
| 1. Ivor Lewis: ti,ab,kw |
| 1. Sweet: ti,ab,kw |
| 1. McKeown: ti,ab,kw |
| 1. Esophagus, Ivor: ti,ab,kw |
| 1. Esophagus, Sweet: ti,ab,kw |
| 1. Esophagus,McKeown: ti,ab,kw |
| 1. Esophageal, Ivor Lewis: ti,ab,kw |
| 1. Esophageal, Sweet: ti,ab,kw |
| 1. Esophageal, McKeown: ti,ab,kw |
| 1. Esophageal Squamous Cell Carcinoma, left, right: ti,ab,kw |
| 1. Esophageal Carcinoma, left, right: ti,ab,kw |
| 1. Ivor-Lewis, Sweet : ti,ab,kw |
| 1. Esophagectomy, Ivor Lewis : ti,ab,kw |
| 1. Esophagectomy, Sweet: ti,ab,kw |
| 1. Esophagectomy,McKeown: ti,ab,kw |
| 1. Esophagectomy, left, right: ti,ab,kw |
| 1. 13 OR 14 OR 15 OR 16 OR 17 OR 18 OR 19 OR 20 OR 21 OR 22 OR 23 OR 24 OR 25 OR 26 OR 27 OR 28 OR 29 OR 30 OR 31 OR 32 OR 33 |
| 1. Cohort Studies: ti,ab,kw |
| 1. Observation:ti,ab,kw |
| 1. Prospective Studies:ti,ab,kw |
| 1. Longitudinal Studies: ti,ab,kw |
| 1. Observational Study: ti,ab,kw |
| 1. Follow-Up Studies: ti,ab,kw |
| 1. Case-Control Studies: ti,ab,kw |
| 1. Cohorts: ti,ab,kw |
| 1. Cohort: ti,ab,kw |
| 1. Cohort Studies: ti,ab,kw |
| 1. Concurrent Study: ti,ab,kw |
| 1. Incidence Studies: ti,ab,kw |
| 1. Observation: ti,ab,kw |
| 1. Prospective Studies: ti,ab,kw |
| 1. Prospectively: ti,ab,kw |
| 1. Prospective: ti,ab,kw |
| 1. Longitudinal: ti,ab,kw |
| 1. Observational: ti,ab,kw |
| 1. Follow-Up: ti,ab,kw |
| 1. Nested: ti,ab,kw |
| 1. Relative risk: ti,ab,kw |
| 1. Hazard ratio: ti,ab,kw |
| 1. RR: ti,ab,kw |
| 1. HR: ti,ab,kw |
| 1. Randomized controlled trial: ti,ab,kw |
| 1. RCT: ti,ab,kw |
| 1. Controlled clinical trial: ti,ab,kw |
| 1. Random*: ti,ab,kw |
| 1. Placebo*: ti,ab,kw |
| 1. Group*: ti,ab,kw |
| 1. Control*: ti,ab,kw |
| 1. 35 OR 36 OR 37 OR 38 OR 39 OR 40 OR 41 OR 42 OR 43 OR 44 OR 45 OR 45 OR 46 OR 47 OR 48 OR 49 OR 50 OR 51 OR 52 OR 53 OR 54 OR 55 OR 56 OR 57 OR 58 OR 59 OR 60 OR 61 OR 62 OR 63 OR 64 OR 65 |
| 1. 12 AND 34 AND 66 |

Note: Search included PUBMED, EMBASE databases, Web of science and Cochrane Library Search strategy：search date was from the inception to March, 2025


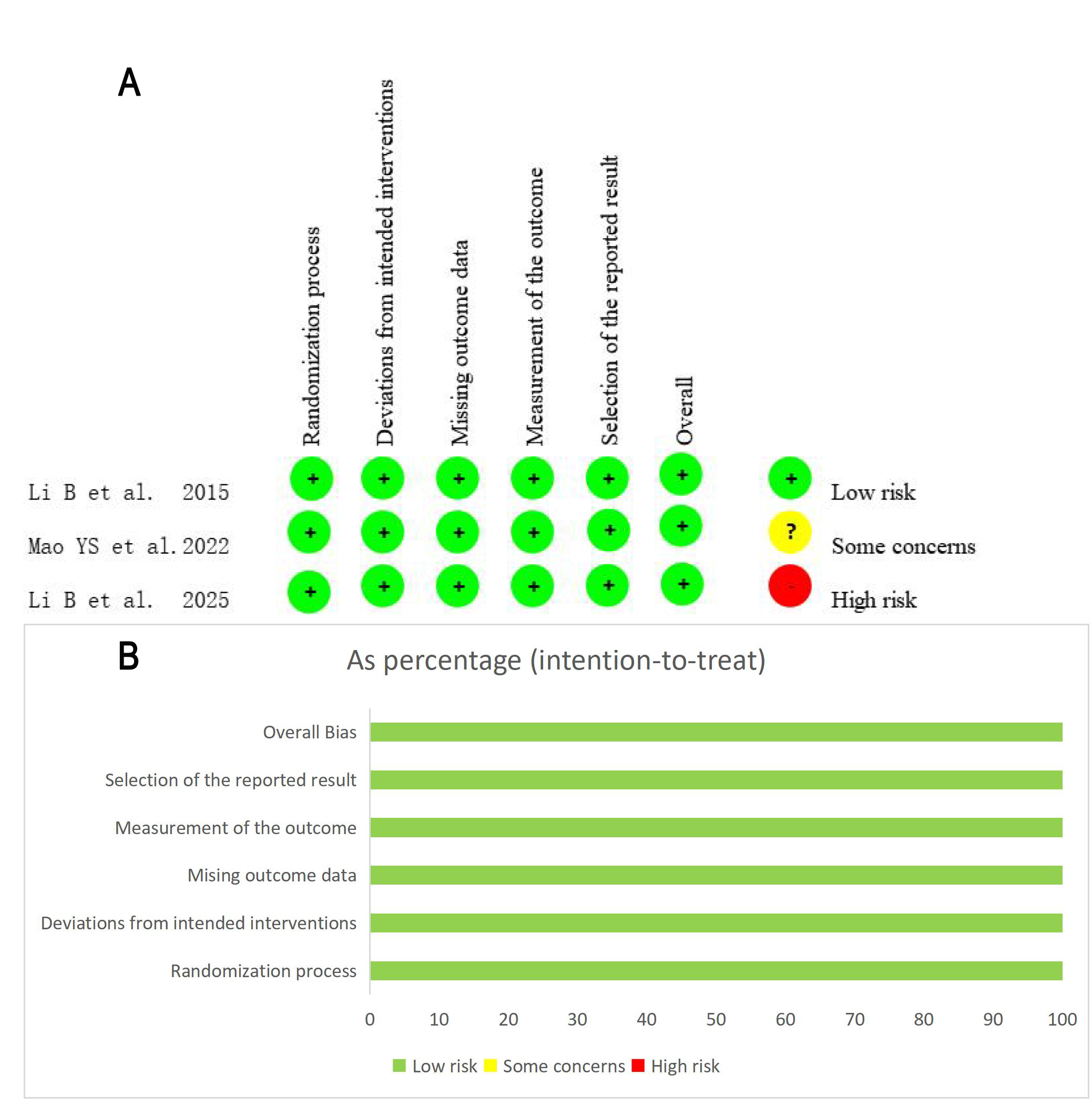


**Supplementary Figure 1** Risk-of-bias assessment result.

**A.**


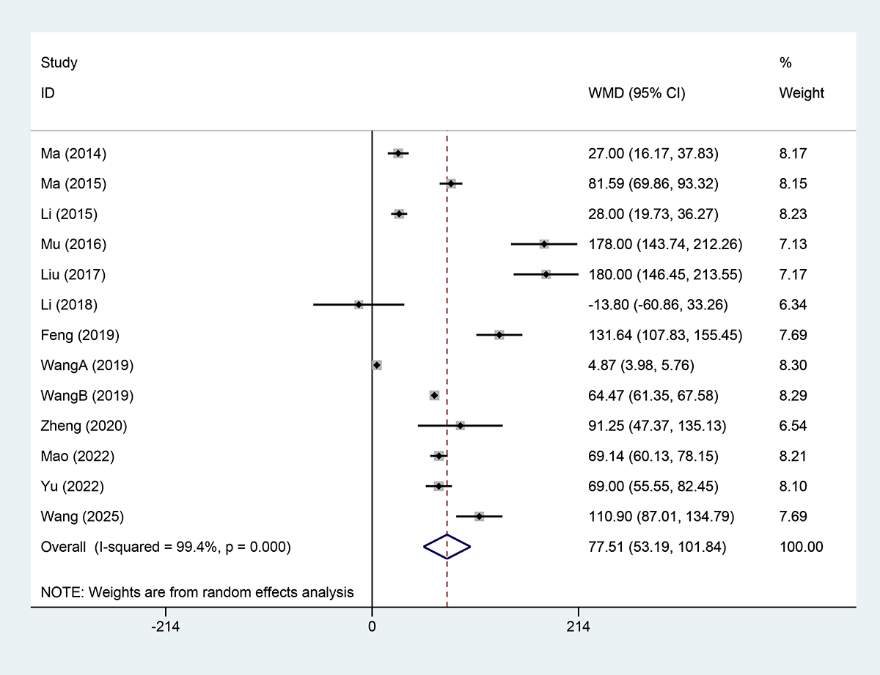


**B.**


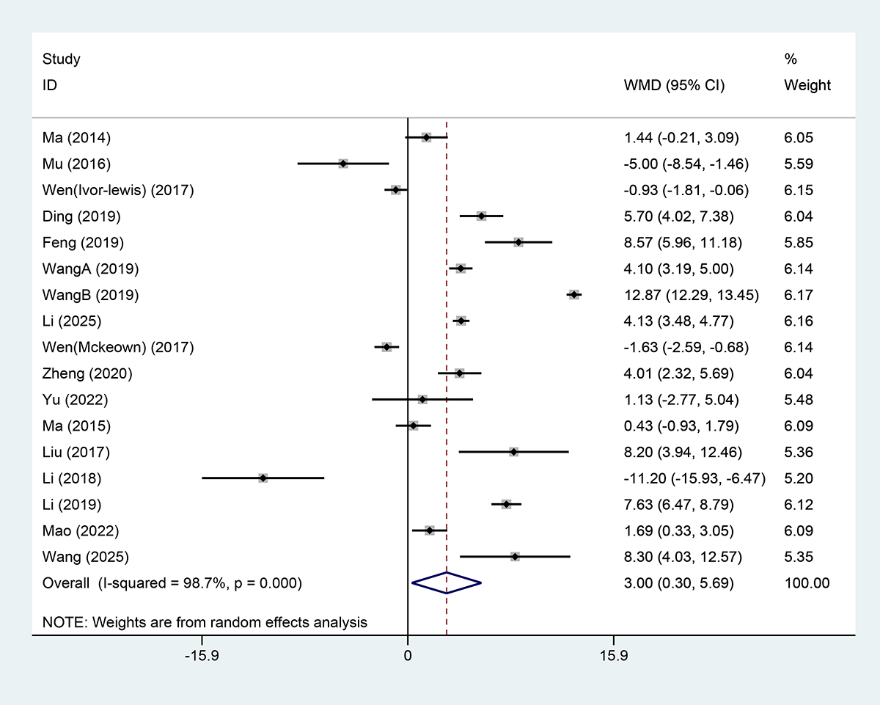


**C.**


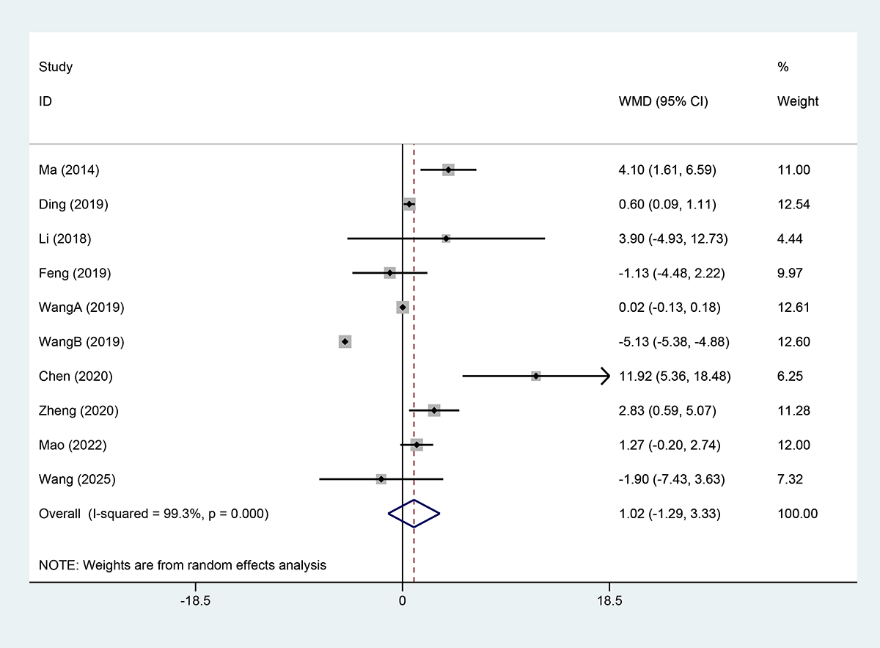


**D.**


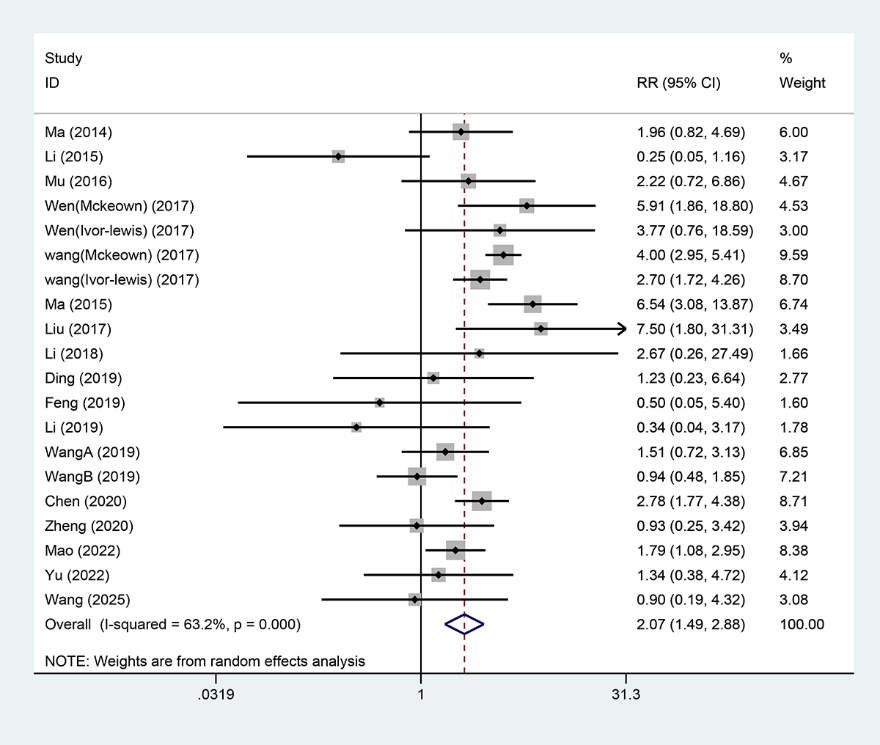


**E.**


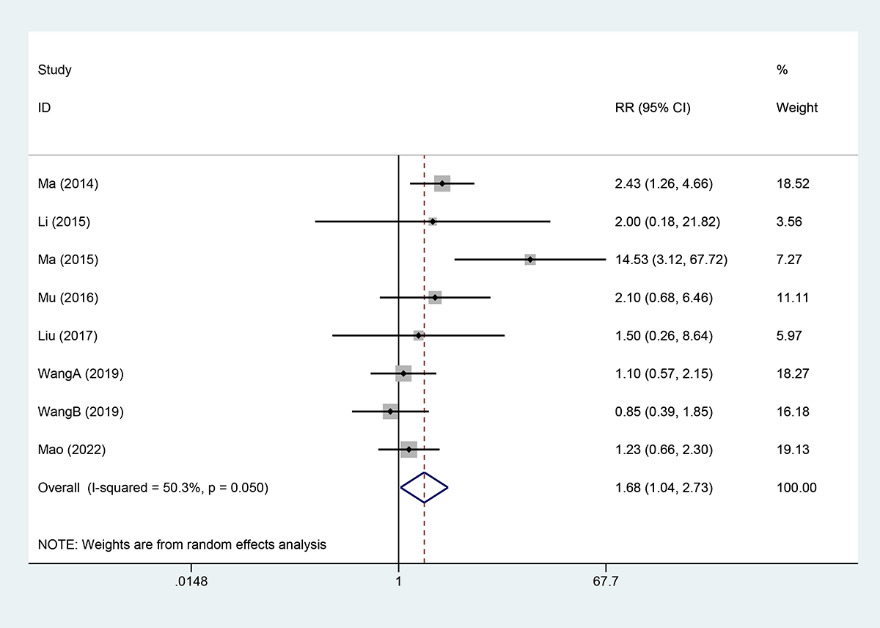


**F.**


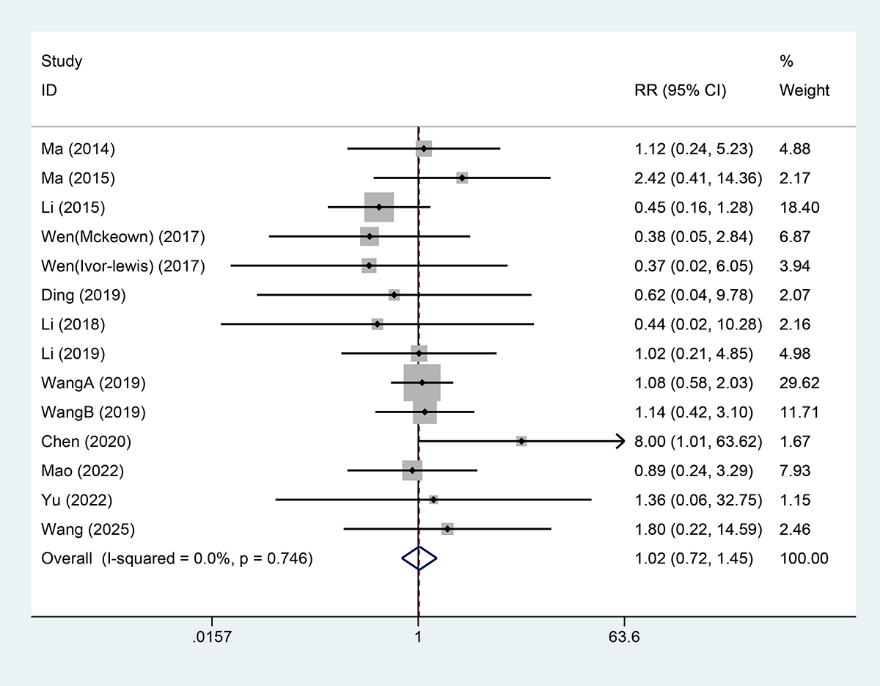


**G.**


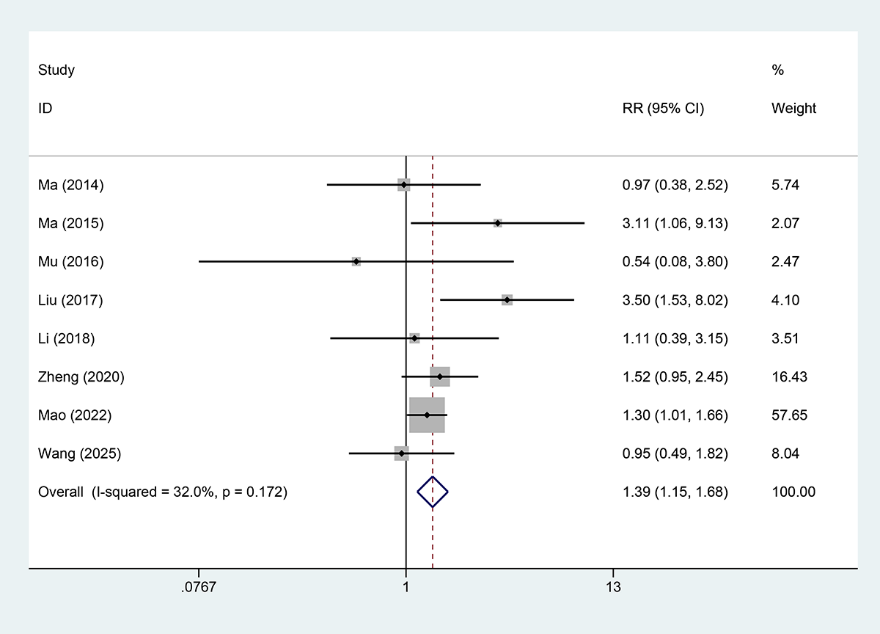


**H.**

**
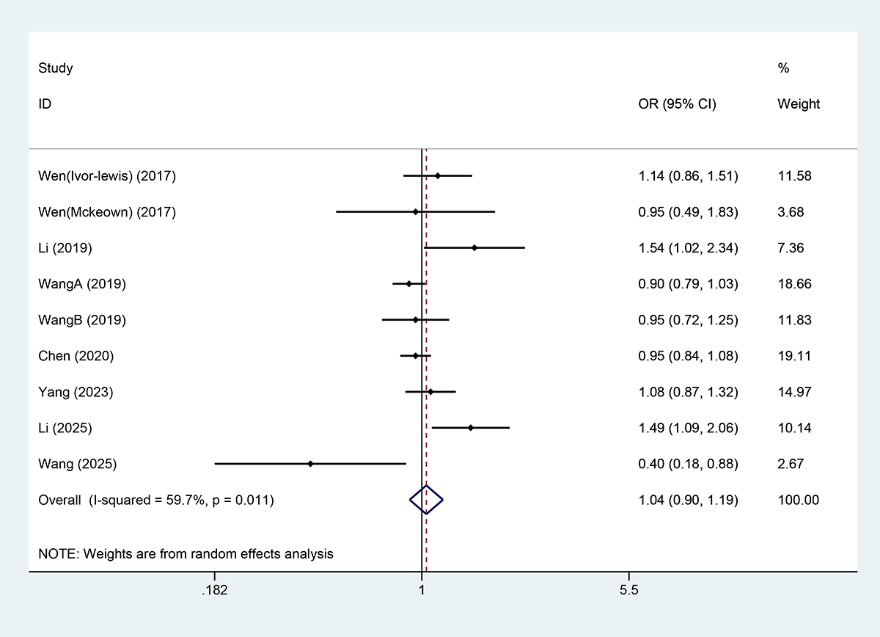
**

**I.**

**
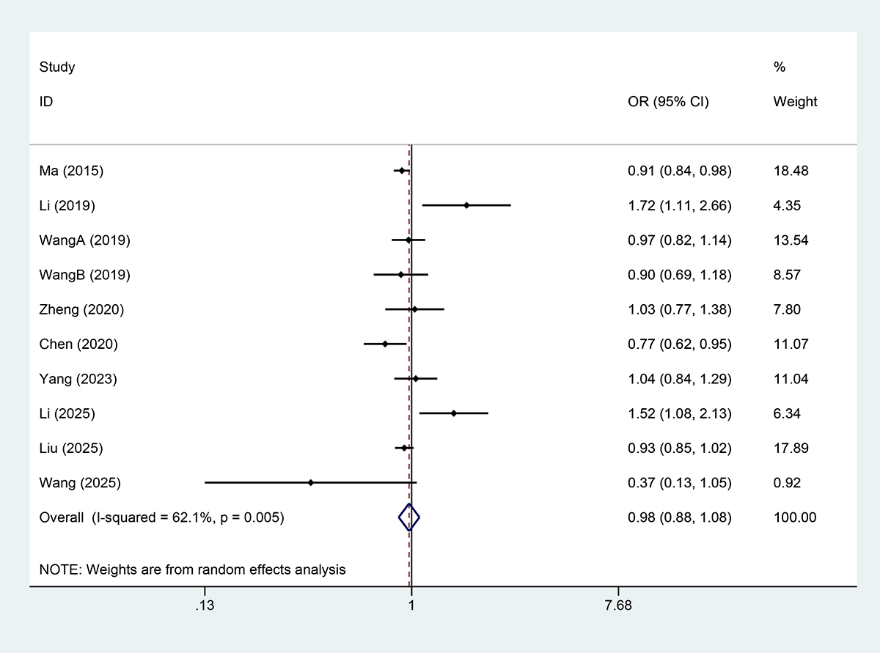
**

**Supplementary Figure 2** Forest plots.

A.Forest plot of operative time; B. Forest plot of lymph node dissection; C. Forest plot of postoperative hospitalization days; D. Forest plot of anastomotic leakage; F. Forest plot of chylothorax; G. Forest plot of pulmonary complications; H. Forest plot of disease-free survival; I. Forest plot of overall survival.

**A.**

**
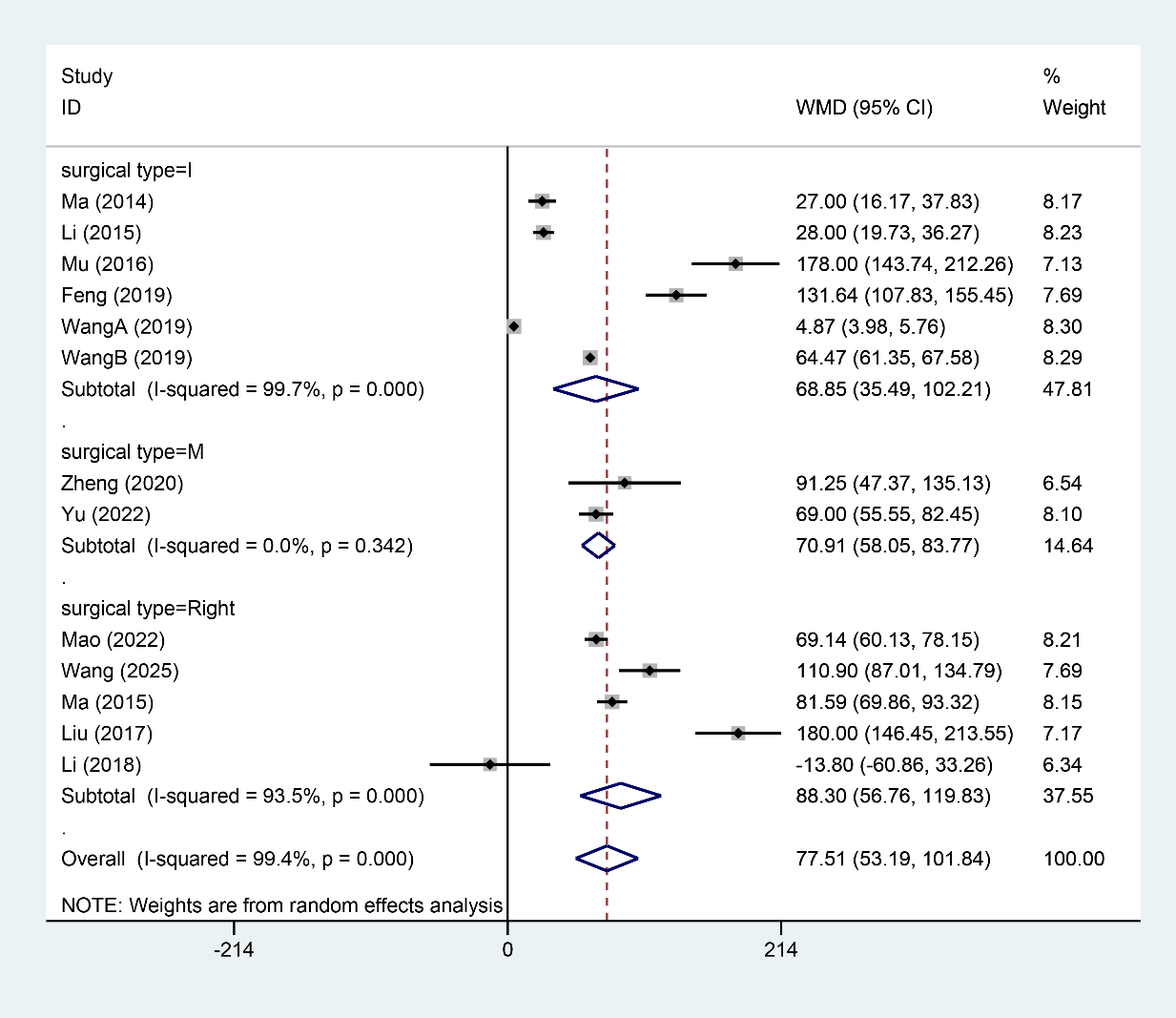
**

**B.**


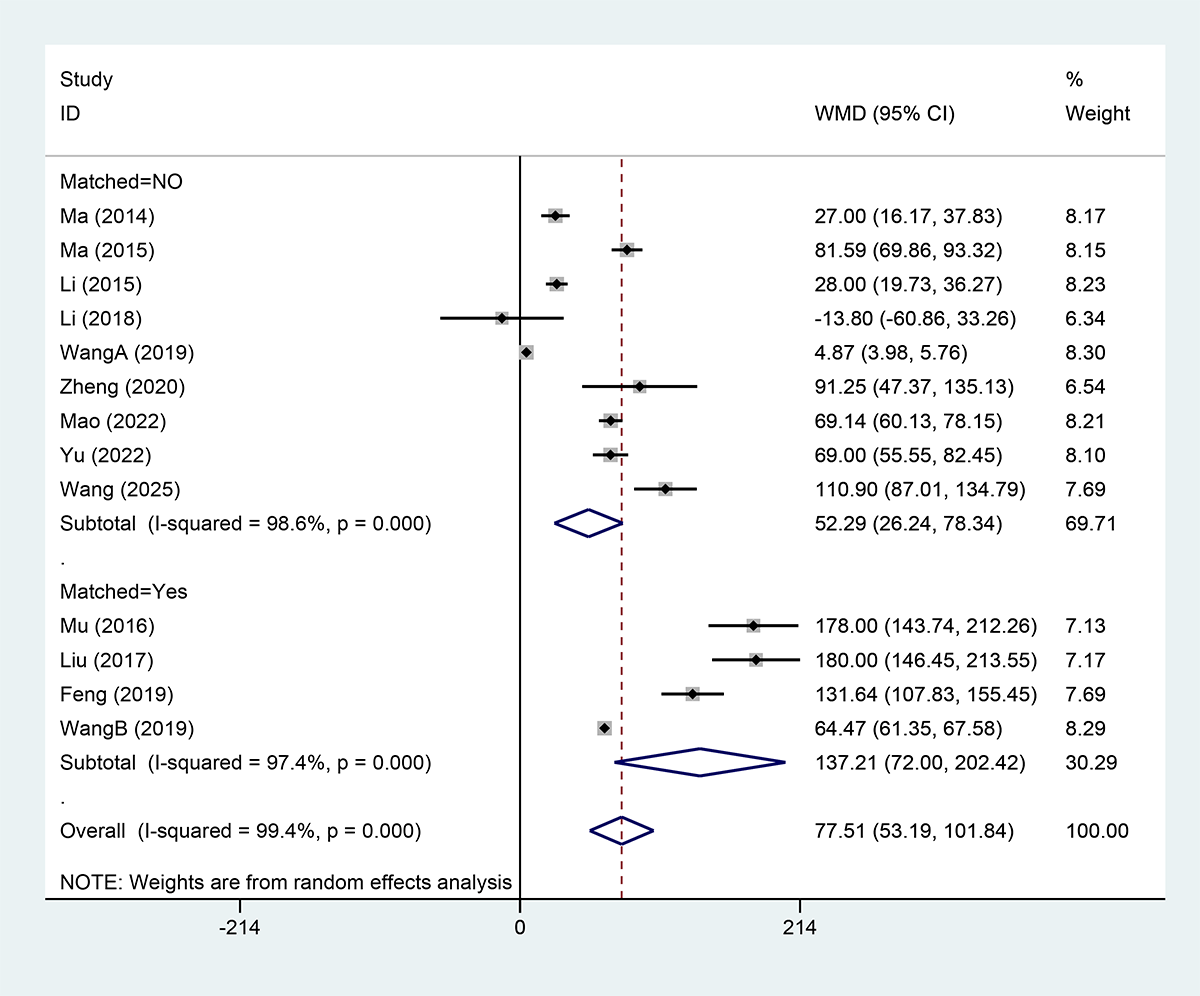


**C.**

**
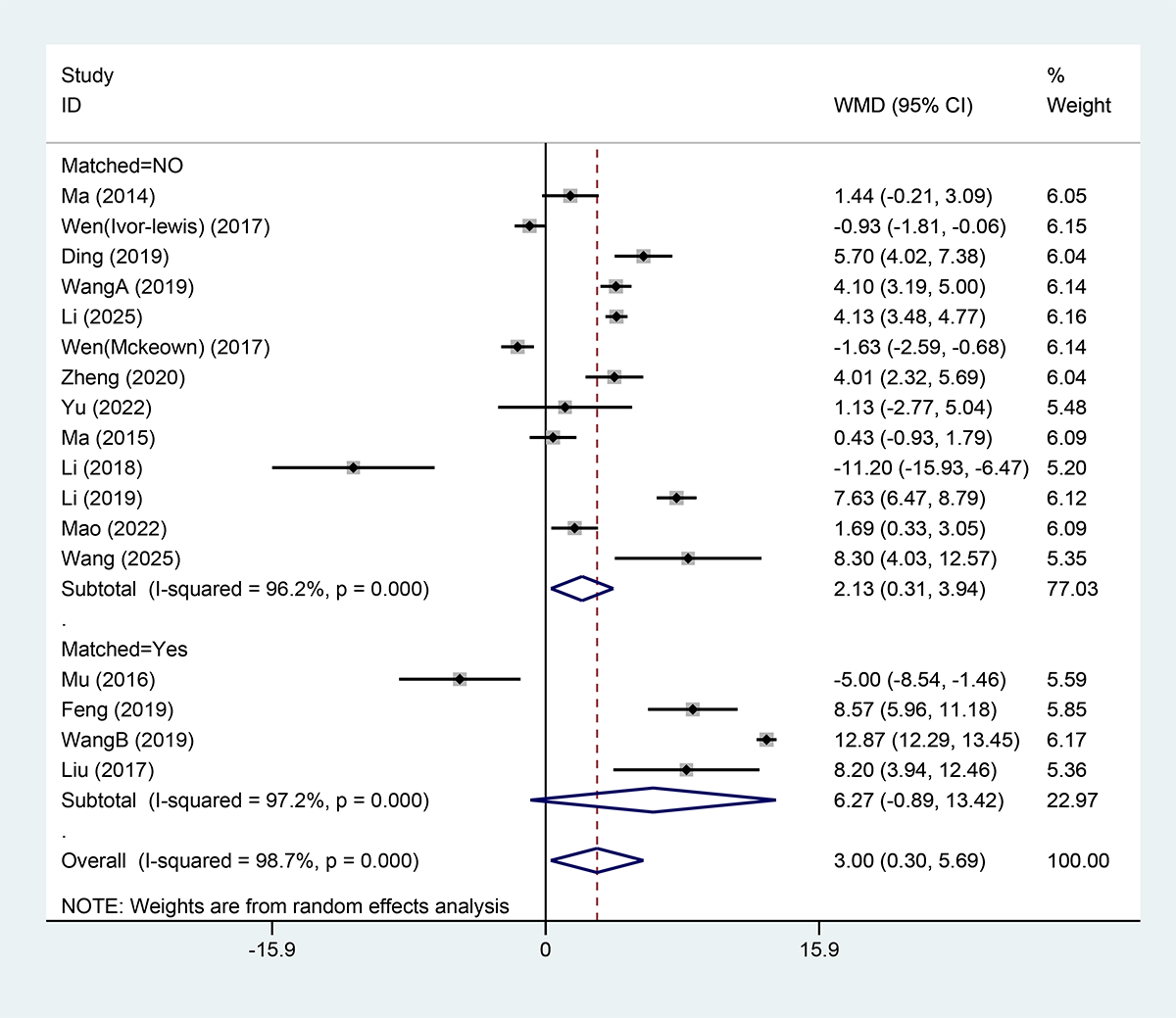
**

**D.**

**
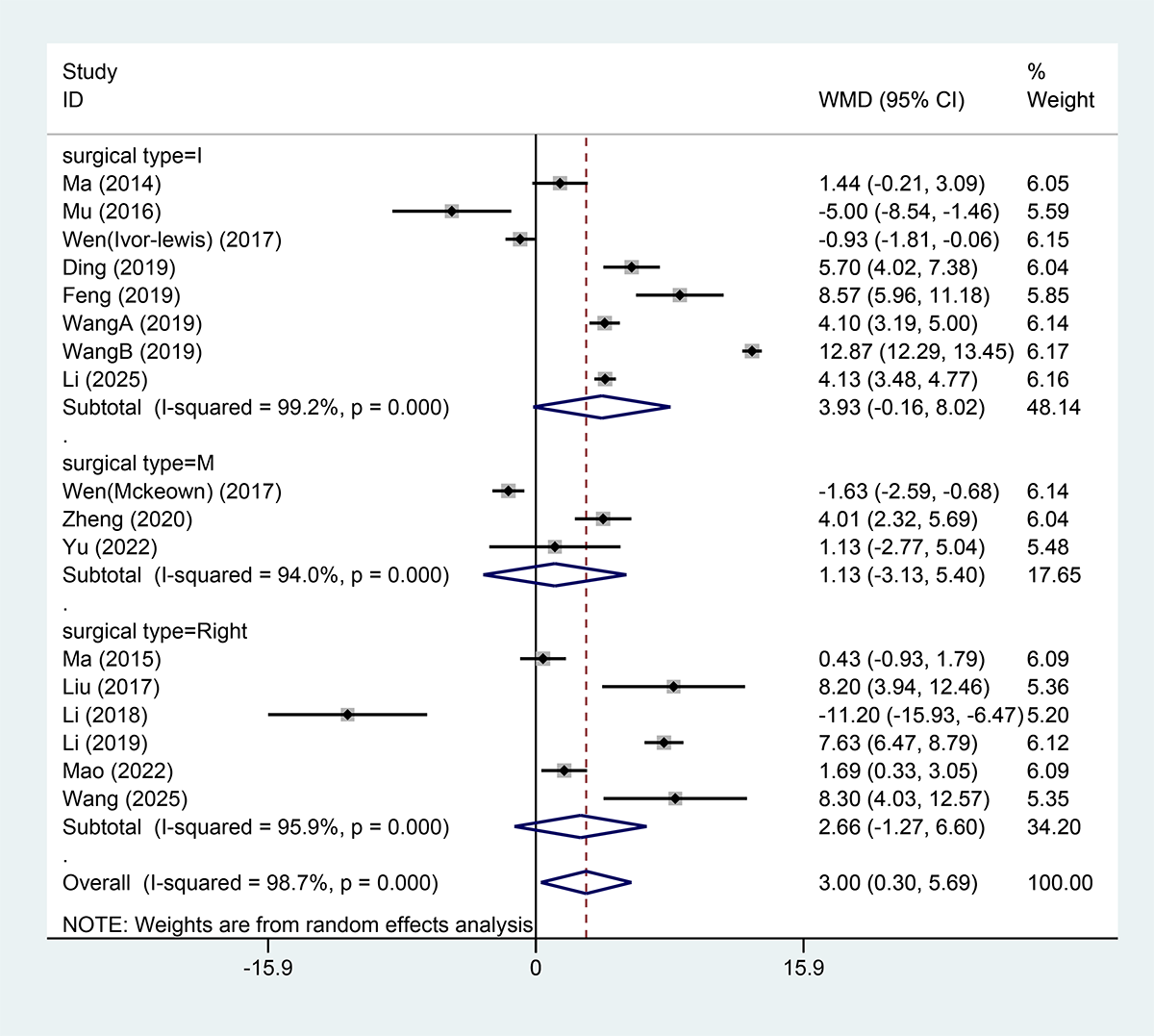
**

**E.**

**
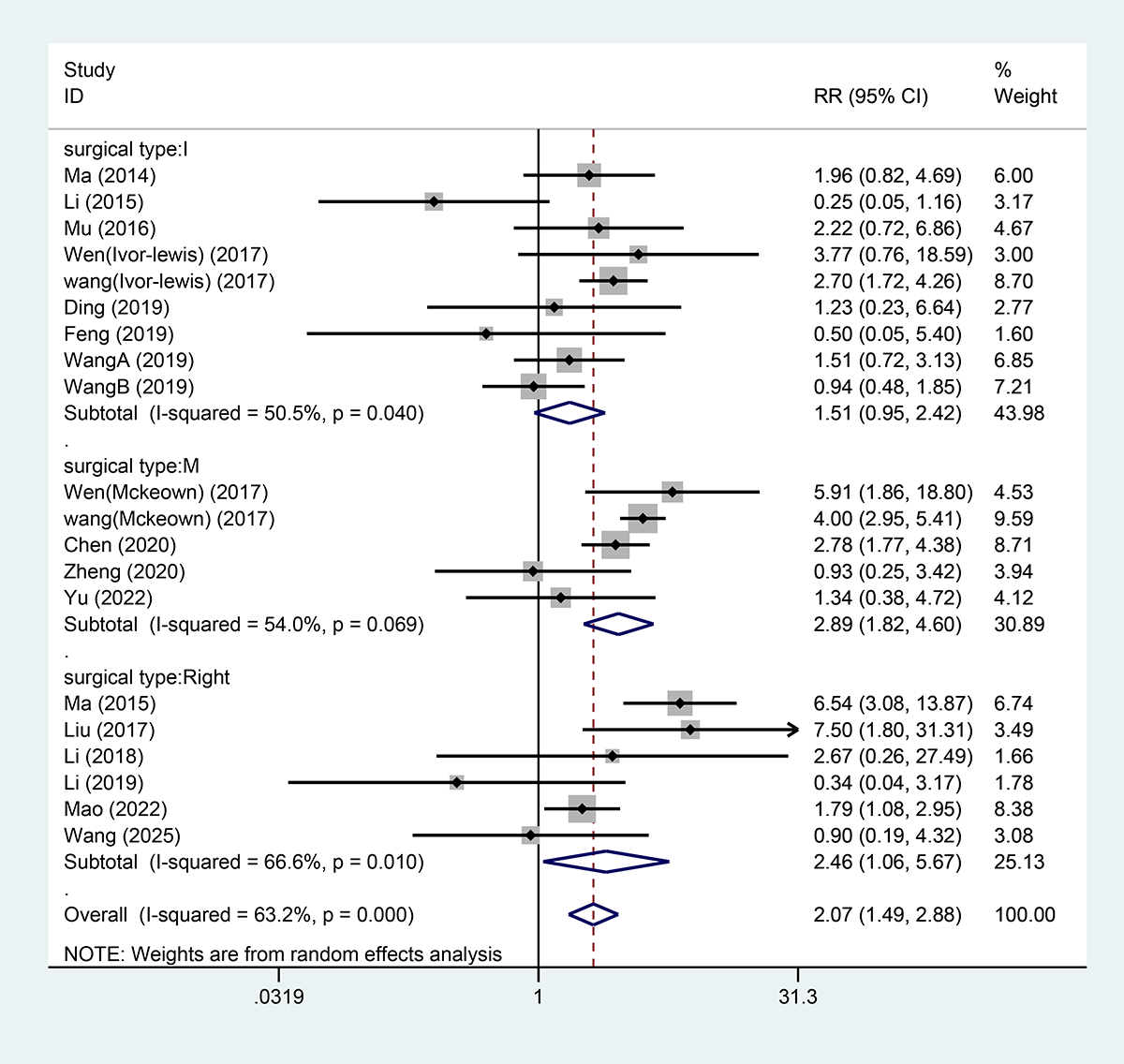
**

**F.**


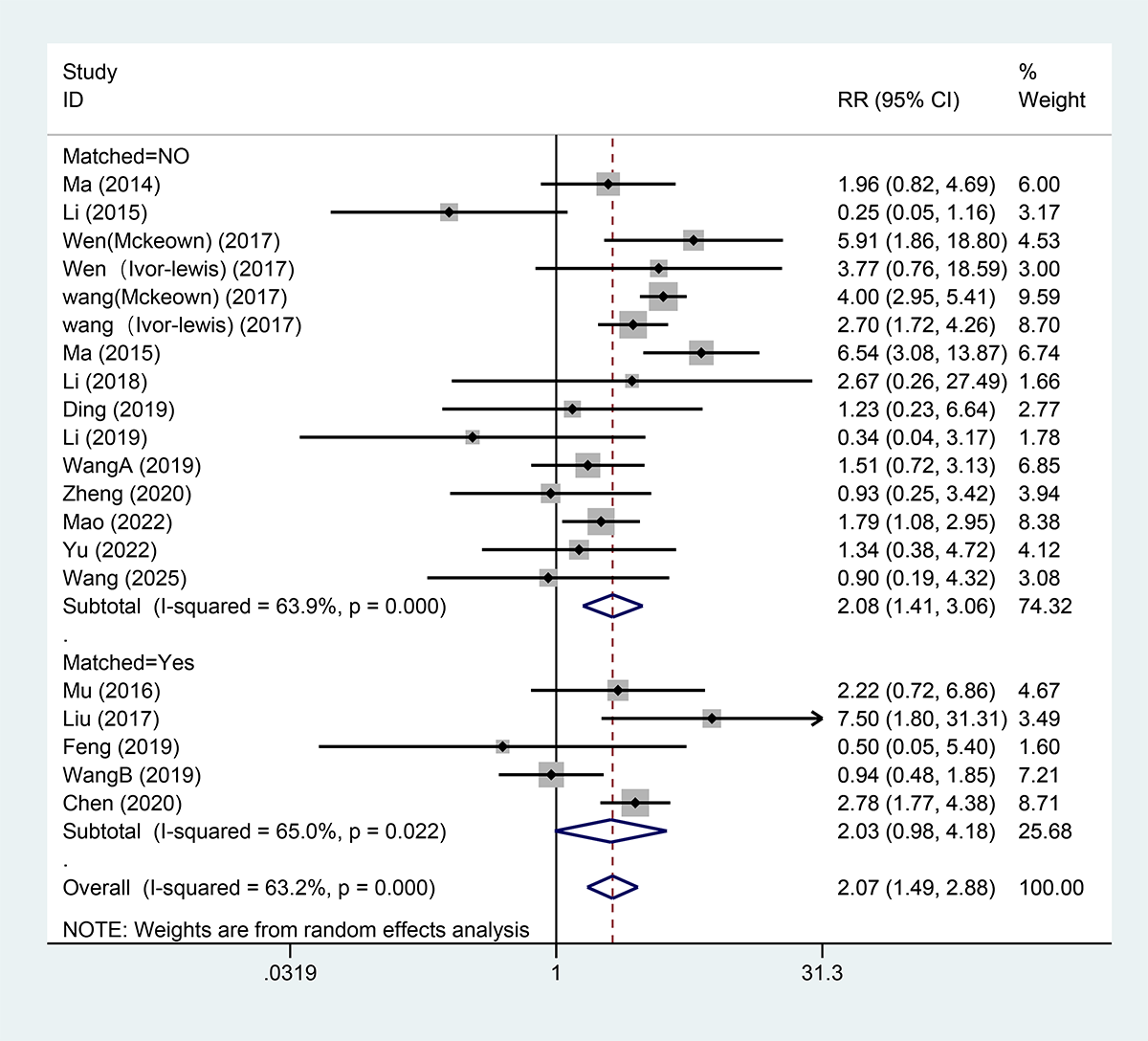


**G.**


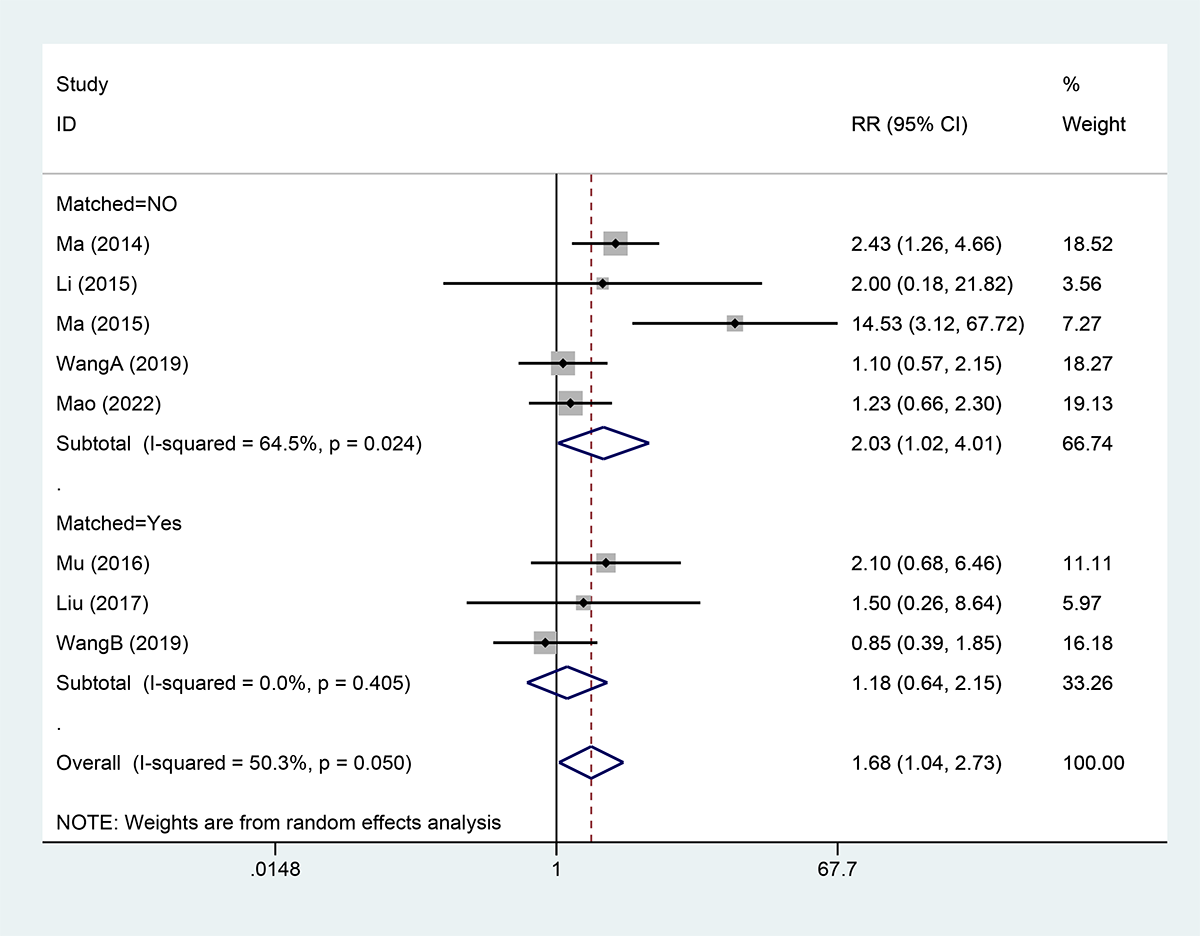


**H.**

**
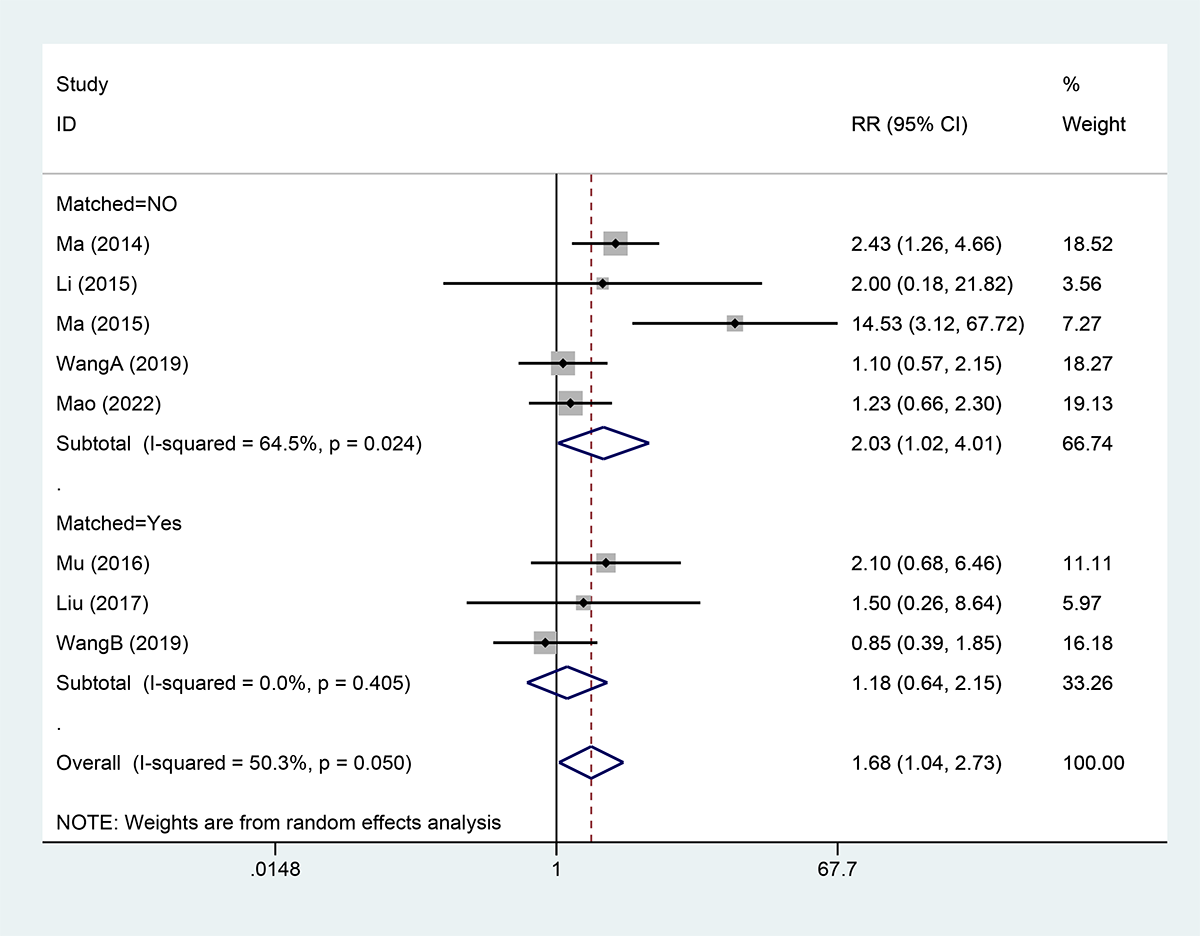
**

**I.**

**
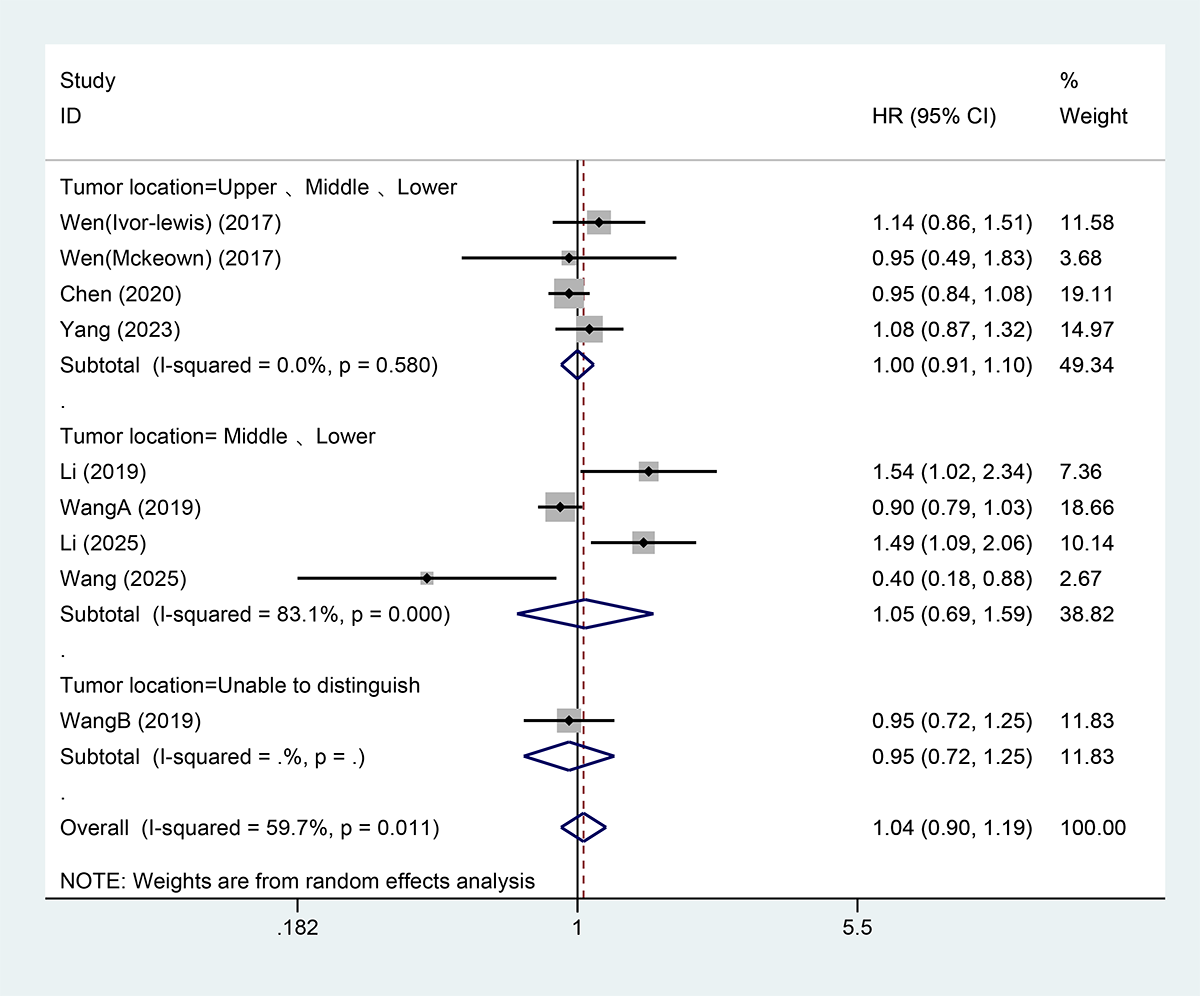
**

**J.**


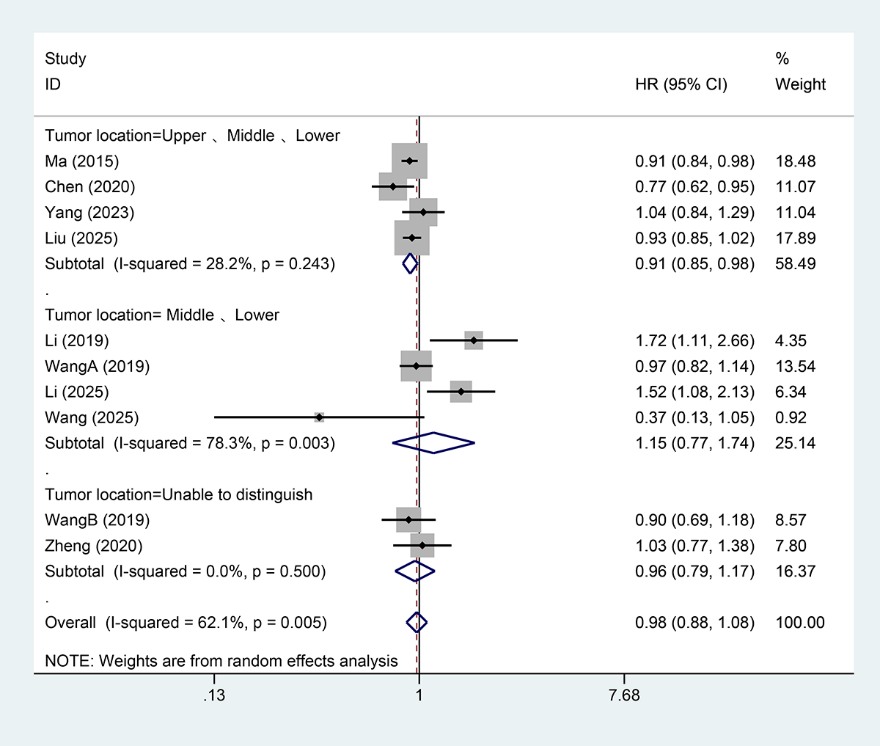


**Supplementary Figure 3** Subgroup analysis chart.

A and B. Subgroup analysis chart of operative time; C and D. Subgroup analysis chart of lymph node dissection; E and F. Subgroup analysis chart of anastomotic leakage; G and H. Subgroup analysis chart of wound infection; I. Subgroup analysis chart of disease-free survival; J. Subgroup analysis chart of overall survival.

**A.**


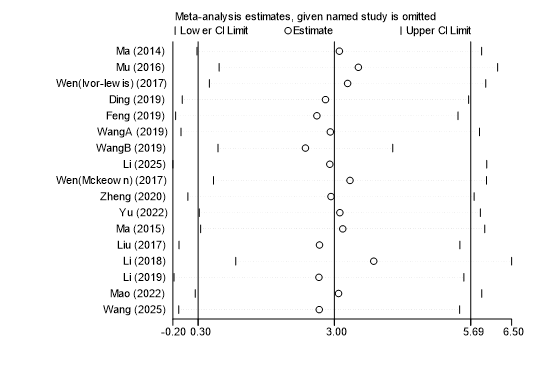


**B.**


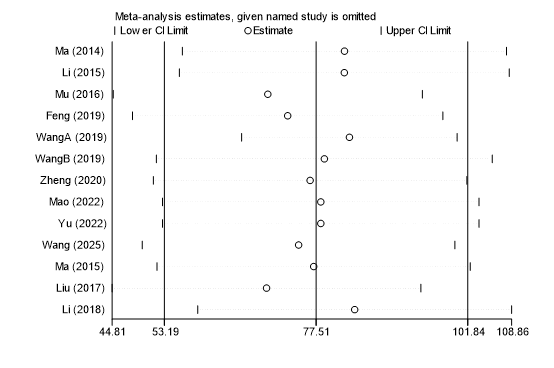


**C.**


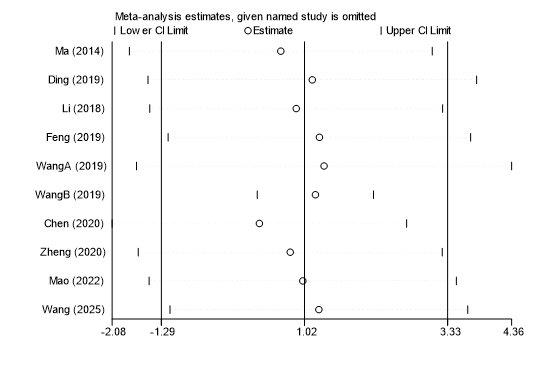


**D.**


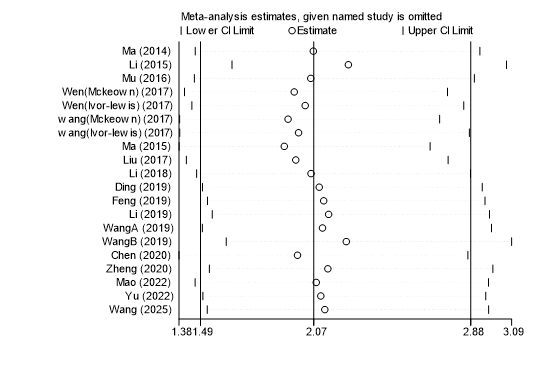


**E.**


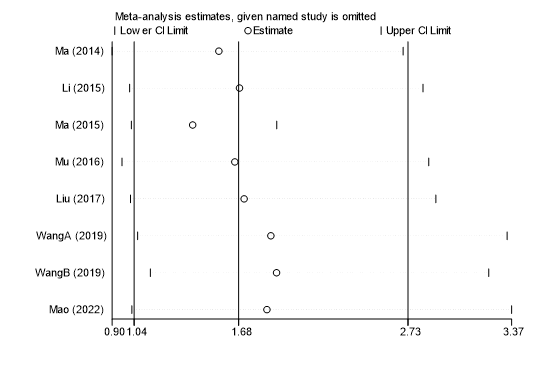


**F.**


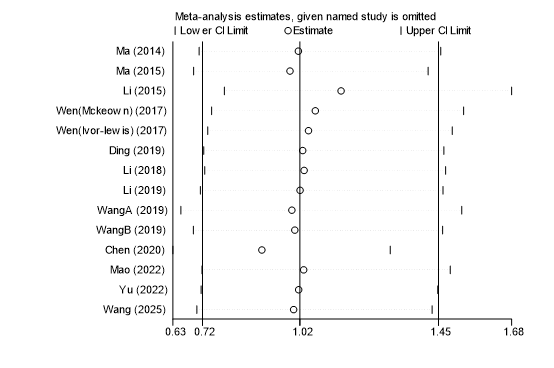


**G.**


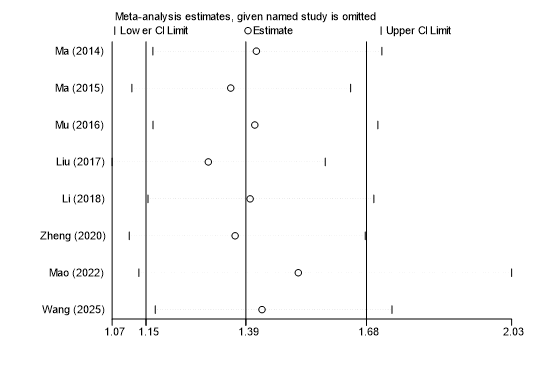


**H.**


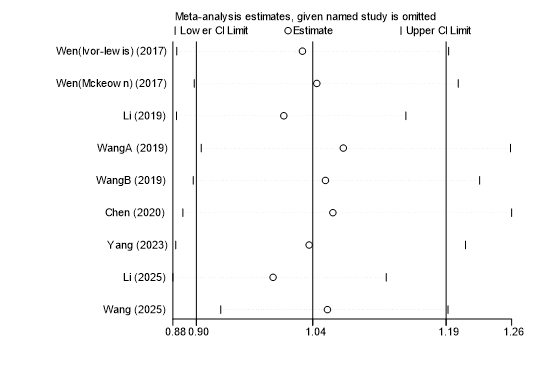


**I.**


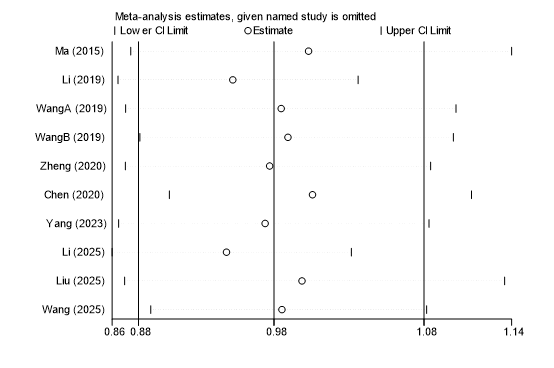


**Supplementary Figure 4** Sensitivity analysis.

A. Sensitivity analysis of lymph node dissection; B. Sensitivity analysis of operative time; C. Sensitivity analysis of postoperative hospitalization days; D. Sensitivity analysis of anastomotic leakage; E. Sensitivity analysis of wound infection; F. Sensitivity analysis of chylothorax; G. Sensitivity analysis of pulmonary complications; H. Sensitivity analysis of disease-free survival; I. Sensitivity analysis of overall survival.

**
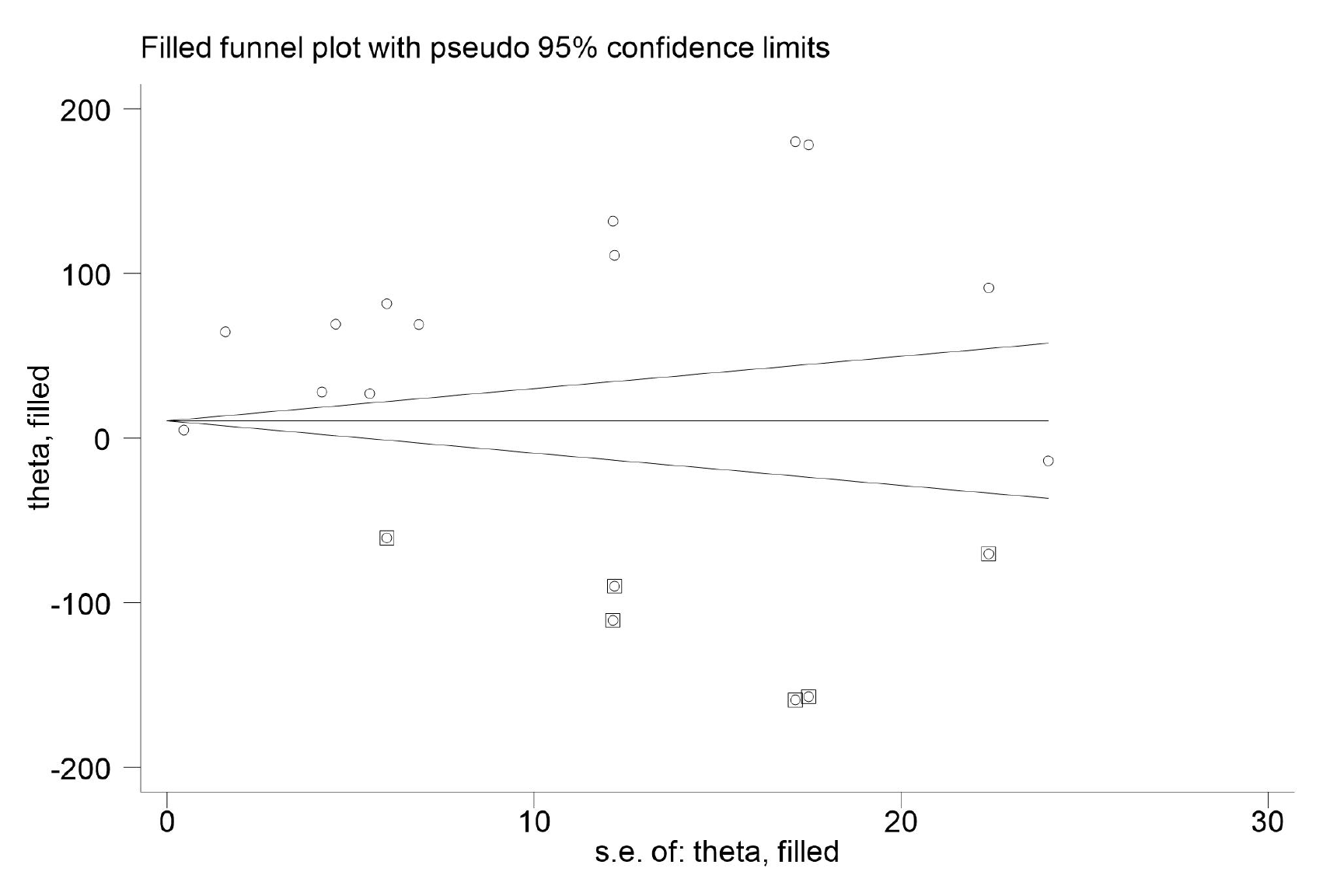
**

**Supplementary Figure 5** Plot of publication-biased Trim-and-Fill analysis of operative time.
